# Morphosyntactic but not lexical corpus-based probabilities can substitute for cloze probabilities in reading experiments

**DOI:** 10.1371/journal.pone.0246133

**Published:** 2021-01-28

**Authors:** Anastasiya Lopukhina, Konstantin Lopukhin, Anna Laurinavichyute

**Affiliations:** 1 Center for Language and Brain, HSE University, Moscow, Russia; 2 Vinogradov Institute of the Russian Language, Moscow, Russia; 3 Independent Researcher, Moscow, Russia; 4 University of Potsdam, Potsdam, Germany; University of Trento, ITALY

## Abstract

During reading or listening, people can generate predictions about the lexical and morphosyntactic properties of upcoming input based on available context. Psycholinguistic experiments that study predictability or control for it conventionally rely on a human-based approach and estimate predictability via the cloze task. Our study investigated an alternative corpus-based approach for estimating predictability via language predictability models. We obtained cloze and corpus-based probabilities for all words in 144 Russian sentences, correlated the two measures, and found a strong correlation between them. Importantly, we estimated how much variance in eye movements registered while reading the same sentences was explained by each of the two probabilities and whether the two probabilities explain the same variance. Along with lexical predictability (the activation of a particular word form), we analyzed morphosyntactic predictability (the activation of morphological features of words) and its effect on reading times over and above lexical predictability. We found that for predicting reading times, cloze and corpus-based measures of both lexical and morphosyntactic predictability explained the same amount of variance. However, cloze and corpus-based lexical probabilities both independently contributed to a better model fit, whereas for morphosyntactic probabilities, the contributions of cloze and corpus-based measures were interchangeable. Therefore, morphosyntactic but not lexical corpus-based probabilities can substitute for cloze probabilities in reading experiments. Our results also indicate that in languages with rich inflectional morphology, such as Russian, when people engage in prediction, they are much more successful in predicting isolated morphosyntactic features than predicting the particular lexeme and its full morphosyntactic markup.

## 1. Introduction

Prediction, as a fundamental aspect of human information processing, has received considerable attention in psychological and psycholinguistic studies in recent years [1, 2]. In psycholinguistics, anticipatory processing of linguistic input has been found in electrophysiological measures [3, 4] and in eye movements during spoken language processing [5, 6] as well as in eye movements registered while reading [7–10]. Psycholinguistic studies on prediction generally use three terms: prediction, predictability, and probability. The umbrella term *prediction* implies that context changes the state of the language processing system before new input becomes available and thus facilitates the processing of this input, see [11]. Predictability and probability are sometimes used as synonyms, e.g., [9] but we will distinguish between them, following other studies [12, 13]. We understand *predictability* as the degree to which a correct prediction about a linguistic unit (e.g., a word, word class, or morphological form) can be made, while *probability* will be understood as a particular estimated numerical measure of predictability.

Predictability of experimental stimuli is a crucial variable in most experiments on prediction in reading, and there are two major approaches to estimate it: human- and corpus-based approaches. Most researchers rely on the human-based approach and use the cloze task [14]. In this task, participants see an incomplete sentence and are asked to produce the most likely next word:

1. *The cause of the accident was a mobile phone*, *which distracted the ______*.

In (1), several nouns are possible, but most people prefer to continue with the word ‘driver’. The cloze probability of the word ‘driver’ in that context is the proportion of times ‘driver’ was produced over all productions. Despite its ubiquitous use, the cloze task has important drawbacks that will be discussed in greater detail in Section 1.1. Studies that rely on the corpus-based approach obtain probabilities from language predictability models [15]. A direct comparison of the two approaches [16–18] provides arguments both in favor of and against substituting cloze probabilities with corpus-based probabilities (see Section 1.1).

One of the major questions about prediction in language comprehension is the nature of the predicted unit [2]. Most experiments investigate *lexical prediction*, namely, the all-or-none activation of a particular word in a particular grammatical form within a sentence [4, 19]. However, lexical prediction is not the only one, and probably a very unusual form of prediction: successful lexical prediction occurs in highly constrained contexts that are rare in natural language [13, 20]. Some studies argue that people can predict incoming information at multiple linguistic levels: morphosyntactic [13, 21], semantic [22], and phonological [23, 24]; see [11] for overview. For example, Luke and Christianson [13] demonstrated that in natural reading, even though people cannot correctly guess the particular lemma, they can predict its word class or its morphological form, and this *partial morphosyntactic prediction* speeds up processing. Therefore, there is a body of evidence that the language processor engages in partial prediction that might occur more often than lexical prediction.

The present study investigates whether the corpus-based approach for measuring lexical and morphosyntactic predictability can provide precise predictability estimates without having the drawbacks of the cloze task. We will compare widely used cloze probabilities with corpus-based probabilities by directly correlating them and by testing, which of them better predicts eye movements in natural reading. Since predictability is a common predictor in many reading experiments, we want to verify that both cloze and corpus-based measures explain a comparable amount of variance in participants’ behavior (namely, fixation durations while reading) and that the explained variance comes from the same source (that is, there is no independent contribution from both cloze and corpus-based measures, and one can safely be substituted by the other). In addition to the traditionally studied lexical predictability we will analyze morphosyntactic predictability, which has more recently come in the spotlight. Exploration of morphosyntactic predictability is particularly important in morphologically rich languages such as Russian, where multiple morphological features can be predicted. Furthermore, our study will be the first to obtain measures for morphosyntactic predictability from corpora, and to compare them to the measures obtained in the cloze task from human participants. We hypothesize that corpus-based probabilities can replace the effortful collection of cloze probabilities in experiments dealing with both lexical and morphosyntactic prediction.

### 1.1. Cloze and corpus-based probabilities

Studies that rely on cloze probabilities as a measure of predictability are based on the following assumption: participants fill in the missing words in the cloze task by sampling from their subjective probability distribution, which remains the same irrespective of whether they engage in production or comprehension. Therefore, cloze probabilities in speech production should be identical to the probabilities that drive participants’ prediction during online comprehension. But recent studies have shown that the cloze task might not be the most precise measure of predictability [18, 25], as probabilities obtained in the cloze task have two major limitations. The first limitation concerns unpredictable words: no probability is available for words that did not come up in the cloze task. Smith and Levy [26] suggested that readers should be highly sensitive to the relative differences in predictability for words within the low predictability range, because the relationship between a word’s predictability and the amount of time taken to read this word is logarithmic. However, low predictability words usually receive zero cloze probabilities in the cloze task and are the ones that are neglected in experiments. As a result, we know very little about how their predictability affects comprehension.

The second limitation is that the answers provided in the cloze task could be systematically lexically biased in a way that makes cloze probabilities an imprecise estimate of predictability. Smith and Levy [18] showed that participants in the cloze task tended to avoid long words, probably to spare the typing effort, and the continuations they provide are shorter than the actual continuations of the sentences. At the same time, participants preferred more formal words to the less formal words that are usually acquired early, perhaps influenced by the formal context of the experiment, which also mismatches the actual continuations of the sentences. Other lexical properties, such as frequency and familiarity, also increase the likelihood that a given word will be produced in the cloze task. Therefore, cloze probability might be a measure of relative activation by context, rather than a measure of predictability, if we equate predictability to conditional probability [25]. Staub and colleagues illustrated the discrepancy between cloze and conditional probabilities with the sentence “*To determine how fast his engine was revving*, *the race car driver checked his __________*” in which the very low frequency word *tachometer* is unlikely to be produced in the cloze task, although many participants might recognize it as the plausible continuation because it is supported by the context.

As an alternative to cloze probabilities, several studies have relied on corpus-based probabilities to predict the variance in reading times and eye movements while reading. In most of these studies, probabilities were produced by classical *n*-gram language models [15]: bigram [27–30], trigram [26], or 5-gram [16, 18] models. These studies do not agree on whether corpus-based probabilities can substitute for cloze probabilities as a measure of predictability. On the one hand, Ong and Kliegl [17] showed for a bigram model and Smith and Levy [18] for a 5-gram model that these models’ probabilities predicted reading times worse than cloze probabilities do. On the other hand, Hofmann and colleagues [16] discovered that corpus-based probabilities from a 5-gram model and a recurrent neural network explained more variance in single fixation durations (SFD, the amount of time a word was fixated, if it was fixated exactly once) than cloze probabilities.

Although n-gram models are widely used in studies of corpus-based probabilities, these models rely only on local context, usually one to four words before the target word. Unlike n-gram models, participants in the cloze task can rely on the entire preceding context and are sensitive to long-distance dependencies induced by syntax and semantics. The inability of n-gram models to take these dependencies into account might explain their relatively poor performance. Recurrent neural network models fare better than n-gram models because they rely on the full sentence context regardless of its length, and make better use of the training data. Presumably, they should also perform more similarly to humans. In language modeling in particular, researchers often choose long-short-term-memory (LSTM) recurrent neural network models [31], which can process long contexts and capture long-distance dependencies [32]. For example, probabilities obtained from LSTM language models were successfully used to predict reading times [33, 34]. We expect that both language models can overcome the above-mentioned biases of the cloze task, as they assign probabilities even to very low predictability words, rely solely on the context, and are not affected by frequency, familiarity or any other lexical properties of words. In this study, we will first demonstrate that the LSTM is a better predictability model than the 5-gram, and then investigate whether the LSTM predicts particular word forms as well as morphosyntactic features of words in sentences similarly to humans.

### 1.2. Lexical and morphosyntactic predictability

Most previous research has investigated the influence of lexical predictability (i.e., the activation of a particular word in a particular grammatical form) on language processing: eyetracking experiments typically compare processing of a word in highly constrained or low constrained contexts. For example, Rayner, Slattery, Drieghe, and Liversedge [35] manipulated the predictability of a target word by changing the preceding context: “*Beth loves performing in front of the camera*. *She wants to be an actress when she grows up*. */ Beth is my best friend’s youngest daughter*. *She wants to be an actress when she grows up*.” In the first example, the target word (“actress”) is more predictable than in the second example. They found that readers looked at less predictable words longer than at highly predictable words. It was also shown that predictability decreased single fixation duration (SFD), first fixation duration (FFD; the duration of the first fixation on the word), gaze duration (GD; the sum of all fixations on a word before the eyes left the word), and total reading time (TT; the sum of all fixations on the word).

Recent studies have shown that people rely not only on lexical predictability, but also on the predictability of some morphosyntactic features of a word. Luke and Christianson [36] provided evidence that readers predict the past tense suffixes in English regular verbs even though they do not predict the lemma of the verb. In another study, Luke and Christianson [13] showed that participants predicted word classes, number marking in nouns, and tense in verbs while reading. A facilitative effect of morphosyntactic probabilities was found in single fixation duration, gaze duration, and total reading times. Crucially, higher morphosyntactic probabilities speeded up reading times above and beyond the words’ lexical probabilities. All these findings indicate that the language processor predicts several morphosyntactic features of words, and that this information facilitates processing.

Notably, most studies on morphosyntactic predictability have been conducted in English, which has relatively poor inflectional morphology [37]. Therefore, experiments that investigate prediction in English can focus only on a very limited number of morphological features that are expressed within a word, such as number in nouns, and number and tense in verbs. In many languages (see https://wals.info/feature), nouns and verbs carry overt inflectional markers, including case in nouns, and gender and person in verbs. These morphological categories could also be predictable in context. For example, in the Russian translation of the example (1) *The cause of the accident was a mobile phone*, *which distracted the ______*, native Russian speakers would predict the accusative case on the upcoming noun *driver*. Higher predictability of case in nouns, and gender and person in verbs, might also facilitate processing. This assumption can be tested for Russian, which expresses case, number, gender, and person morphological categories within a word [38].

### 1.3. The present study

This study investigates whether corpus-based probabilities obtained from the LSTM language model can replace cloze probabilities. Our main goal is to test whether corpus-based probabilities are as good as cloze probabilities (or can outperform the latter) in explaining variance in fixation durations during reading. We will focus on four eyetracking measures: single fixation duration (SFD), first fixation duration (FFD), gaze duration (GD), and total reading time (TT). If cloze and corpus-based probabilities strongly correlate and explain the same variance in fixation durations, the corpus-based probabilities could replace the cloze probabilities in psycholinguistic experiments.

We evaluate two major types of predictability language models: the 5-gram model and the LSTM recurrent neural network, both trained on three large corpora of Russian. We will show that the LSTM is generally a better predictability model than the 5-gram model.

Our central goal is to compare the well-studied lexical predictability measures obtained from the cloze task and those of the LSTM model. Cloze probabilities should reflect relative activation of a word in context, whereas corpus-based probabilities estimate the conditional probability of a word. Therefore, these two types of probabilities should not be identical. But if corpus-based probabilities strongly correlate with cloze, and both types of probabilities explain the same variance in eye movements while reading, it will mean that not only cloze, but also corpus-based probabilities can reflect comprehenders' subjective probability estimates. That could allow researchers to use corpus-based probabilities instead of cloze probabilities when they aim to study or control for probabilities.

Furthermore, we will explore morphosyntactic prediction during reading in the morphologically rich Russian language. We will estimate whether word class and morphological predictability influence eye fixation durations beyond lexical predictability. Following Luke and Christianson [13], we hypothesize that word class, number in nouns, and tense in verbs will be predictable in context and will influence reading speed. Additionally, we want to explore whether gender and case in nouns as well as person, number, and gender in verbs might be predictable and facilitate processing. Crucially, for the first time we will compare cloze and corpus-based morphosyntactic probabilities. If the LSTM model produces corpus-based morphosyntactic probabilities that explain the same variance in fixation durations as probabilities collected from participants in the cloze task, then corpus could substitute for cloze both in lexical and morphosyntactic probability estimations.

## 2. Language predictability models

### 2.1. Specification of the models

We compare two main kinds of language predictability models: an n-gram model and a recurrent neural network model. The 5-gram model with Kneser-Ney smoothing [39] is implemented in SRILM v1.7.2 Toolkit [40]. The long-short-term-memory (LSTM) recurrent neural network language model is a one layer LSTM-2048-512 from Jozefowicz, Vinyals, Schuster, Shazeer, and Wu [41] that has performed well for language modeling on large corpora. The size of the hidden state is 2048; the size of the input and output token embeddings is 512. The particular choices of the hidden state and embedding size is a compromise between model accuracy (larger values give a more powerful model) and model size with runtime performance: probabilities can be obtained on a modern laptop in a reasonable amount of time.

Each of the two models was trained separately on three text corpora: the Russian National Corpus (RNC; ruscorpora.ru), the Taiga corpus [42], and the combination of RNC and Taiga. The training part of the RNC consists of 577 million tokens and includes modern written texts from the 1950s to the present day, real-life Russian oral speech recordings from the same period, early written texts from the middle of the 18th to the middle of the 20th centuries, and newspaper articles published in and after the year 2000. The Taiga corpus consists of about 500 million tokens and comprises 33 literary magazines (50% of texts), news from four popular websites (25% of texts), and texts from culture magazines, social networks, and websites with amateur poems and prose (25% of texts). For testing, we randomly selected 1,000 sentences from another corpus: the OpenCorpora (http://opencorpora.org/; [43]). It consists of 1.9 million tokens and includes newspaper articles, Russian Wikipedia, texts from blogs, fictional and non-fictional pieces, and legal documents.

### 2.2. Evaluation metrics

To select the best model, we obtained the models’ perplexity and accuracy. Model accuracy of is the ratio of correctly predicted words to all predicted words. This measure allows us to compare the language models and human answers in the cloze task. However, it does not account for the fact that in many contexts, there are several plausible continuations, and in the accuracy measure, the model that provides a plausible but non-target word would be penalized as much as a model that makes a completely improbable prediction. Perplexity (a standard measure for language model evaluation), gets around this issue by using probabilities. The model assigns probabilities to all words, and for its perplexity evaluation the probability of the target word is used: higher probability leads to lower perplexity; informally, the model is less “surprised” by the target word. However, this measure does not allow us to compare model and human performance, as the cloze task does not provide probabilities for all possible words.

### 2.3. Results

We evaluated the 5-gram and LSTM models trained on three corpora and found that the LSTM model consistently outperformed the 5-gram model: it showed higher accuracy and lower perplexity (see Table 1). All models were tested on 1,000 sentences sampled from OpenCorpora (opencorpora.org); first words in sentences and punctuation were not evaluated. The best results were obtained for the LSTM trained on the Russian National Corpus (RNC), so this model was selected for further studies and produced lexical and morphosyntactic corpus-based probabilities that we then compared with cloze probabilities. The LSTM model trained on the RNC as well as the code for training the model are freely available at https://osf.io/yq46b/ and with interface at http://lm.ll-cl.org/.

**Table 1:** Accuracy (higher is better) and perplexity (lower is better) of the 5-gram and the LSTM models trained on three corpora.

	Training corpus	Accuracy	Perplexity
5-gram model	RNC	0.136	1089
	Taiga	0.126	1260
	RNC + Taiga	0.137	1058
LSTM model	RNC	**0.173**	**328**
	Taiga	0.148	454
	RNC + Taiga	0.169	357

## 3. Correlation of cloze and corpus-based probabilities

### 3.1. Method

#### 3.1.1. Participants

In total, 605 native speakers of Russian participated in the cloze experiment (365 female; mean age = 31, range 18–81 years). All participants were recruited through social networks. They reported that Russian was their only native language.

#### 3.1.2. Materials

All materials were taken from the Russian Sentence Corpus described in detail by Laurinavichyute, Sekerina, Alexeeva, Bagdasaryan, and Kliegl [44]. These are 144 sentences extracted from the Russian National Corpus that have diverse syntactic structures and vocabulary. Sentence length ranges from 5 to 13 words, with an average of 9 words. They contain a total of 1,362 words. These sentences were excluded from the aforementioned training corpus for the language predictability models. The materials are available at https://osf.io/36ckr/.

#### 3.1.3. Procedure

Participants were instructed to perform the cumulative cloze task: they were repeatedly asked to type into the online response box a word that would provide the most natural continuation of the sentence, starting from the first word in the sentence. After each attempt, they saw the actual word in the sentence above the response box and were prompted to make the next guess. They progressed word by word and could not skip words or go back to their previous responses. No restrictions were posed on the number of words and sentences each participant completed. Data from every participant who made guesses for more than 20 words were included. Overall, each word received 20 to 151 guesses, with a mean of 47. We manually corrected misprints and spelling mistakes. The first words of the sentences were excluded from the analyses because the participants had no context for them. Overall, 1,218 words were subjected to the correlation analyses.

#### 3.1.4. Analyses of cloze probabilities

To compute probabilities for every target word, participants’ answers in the cloze task were compared to the target words. Probabilities were logit-transformed; zero cloze probabilities (target words that no participant guessed) were smoothed: zeros were replaced with 1/(2*number of guesses for the word), see [44, 45]. *Lexical probabilities* were computed only for words that matched the target words orthographically and thus provided a full match (e.g., *svidetelej* ‘witnesses’ in genitive plural). *Morphosyntactic probabilities* included word class probability (we distinguished between nouns, verbs in finite forms, infinitives, adjectives, adverbs, numerals, personal pronouns, prepositions, conjunctions, and particles), three morphological features for nouns (number, gender, and case), and four morphological features for verbs (tense, person, number, and gender). Each word that the participants provided in the cloze experiment and each word in the stimuli sentences was tagged for word class and morphological features using the PyMorphy2 analyser [46], and participants’ responses were compared to the target words. A word class match was coded if the predicted and target words belonged to the same word class. Similarly, the morphological matches were coded for gender, number, and case for nouns; and tense, number, person, and gender for verbs. Finally, when the participants predicted the word class (or any other morphological feature) of the upcoming word, the probability of this predicted word class was computed by summing the probabilities of all participants’ responses which had that word class. For example, to estimate the word class probability in a sentence that begins *A mobile ______* where "phone" is the target noun, we add up the probabilities of all nouns in the participants’ responses. For morphologically ambiguous words (e.g., *rot* ‘mouth’ is ambiguous between the nominative and accusative singular), all possible variants were considered in the probability estimation. The cloze task data are available at https://osf.io/yq46b/.

#### 3.1.5. Analyses of corpus-based probabilities

The language model assigned probabilities to all words in the stimuli sentences. These raw corpus-based probabilities were then logit-transformed: 0.5*ln(probability/(1-probability)). Lexical and morphosyntactic probabilities were computed in the same way as in Section 3.1.4. with one distinction: probabilities were estimated for each word in the model’s vocabulary (i.e., the 500,000 most frequent words in the training corpus) and the predictions of the model were compared to the annotation of the target words.

### 3.2. Results

#### 3.2.1. Lexical probabilities

**Fig 1:**
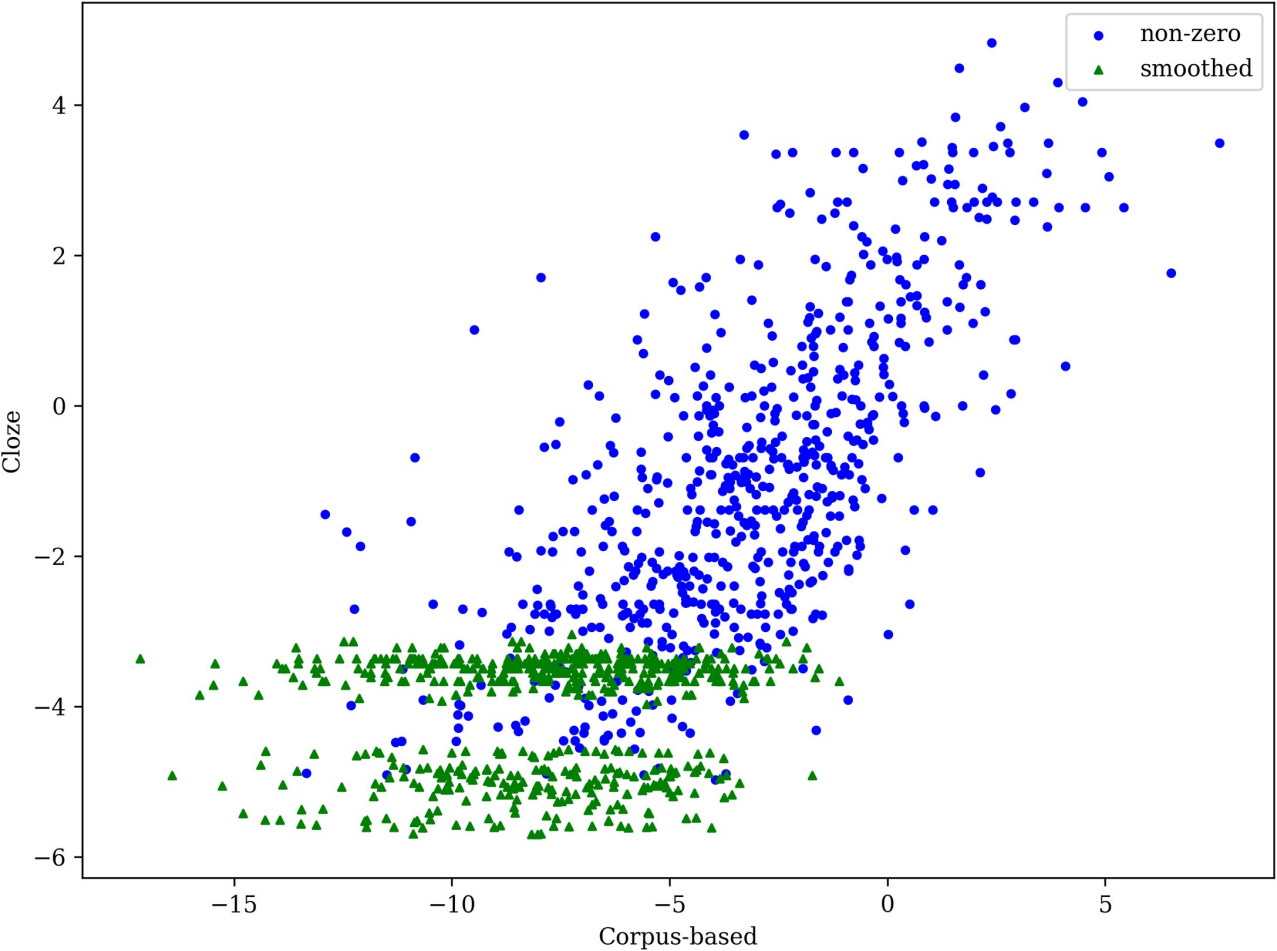
Cloze versus corpus-based probabilities in logit space. Each point represents a single word. Words for which cloze probability is smoothed are shown with green triangle markers; smoothing is used when no participant in the cloze experiment provided a correct guess (567 out of 1,218 total words).

For the words in 144 experimental sentences, the mean probability of correct words obtained from participants in the cloze task was 0.184, while the mean probability from the LSTM language model was 0.195, which is very close. We also analyzed the correlation between cloze and corpus-based probabilities: we took both the Pearson correlation on logit-transformed values and the Spearman correlation that is sensitive only to rank and allows including words with zero probabilities from the cloze task. For our goal, it is important that the language model assigns low probabilities to words that have zero probabilities in the cloze task. The Pearson correlation between the cloze and LSTM logit-transformed probabilities is 0.68 without zero cloze probabilities, or 0.72 with smoothed zero cloze probabilities (see Fig 1), while the Spearman correlation is 0.73. The Pearson correlation in our study is higher than that in the study by Smith and Levy [18], where the highest Pearson correlation was 0.59. We hypothesize that the lower correlation reported by Smith and Levy may be explained by the limitations of the n-gram language model they used and by the context of only four words they provided for prediction. At the same time, we acknowledge that the difference might also be caused by other factors like the language of study (English or Russian), the choice of experimental materials, the training corpora for the models, etc.

**Fig 2:**
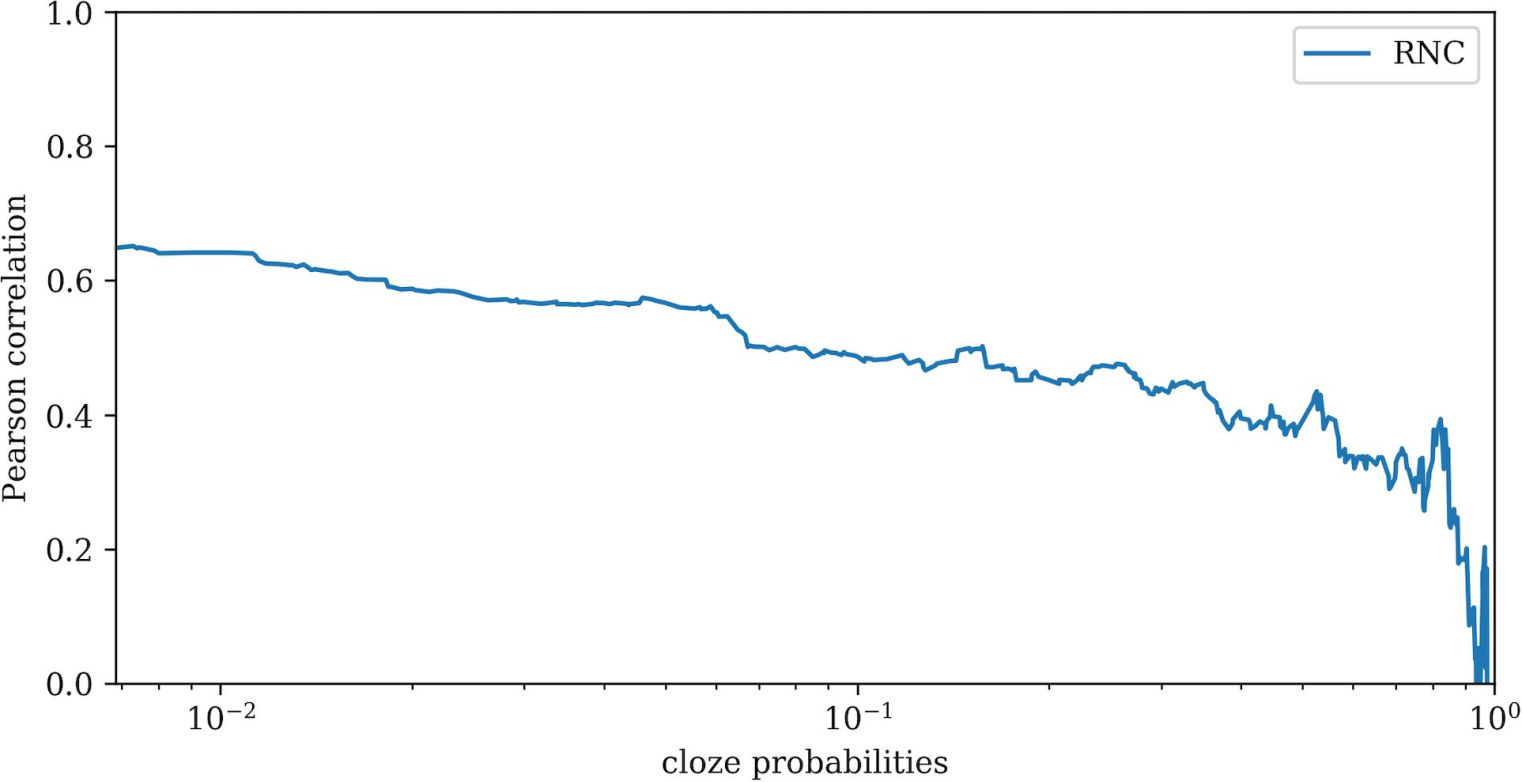
The degree of Pearson correlation on the *y* axis; cloze probabilities on the *x* axis. Pearson correlation is computed for all words with cloze probabilities lower than the probability value on the *x* axis.

We also analyzed how the correlation changes depending on word probability. Fig 2 shows that the Pearson correlation is higher for less predictable words (the left part of the line chart) than for more predictable words (the right part of the line chart). Note that the correlation of answers with zero cloze probabilities is not plotted. The analysis revealed, firstly, that the LSTM model systematically assigned lower probabilities, secondly, that overall correlation was high, and thirdly, that correlation is slightly lower for words that are highly predictable by humans in the cloze task.

To gain insights about the nature of the discrepancy between the cloze and corpus-based probabilities, we qualitatively analyzed 53 highly predictable words in the cloze task (those with close scores higher than 0.7; the mean was 0.84) that obtained low corpus-based probabilities (lower than 0.3; mean = 0.14). In about one third of the cases, the LSTM model assigned the highest probability to the target word, but the model also provided many variants as plausible continuations of the sentence, and, as a result, the target word got a low probability score. For example, in the sentence *Jemu udalos’ vskryt’ banku ob ostryj kraj bampera svoego _________* ‘He managed to open the can with a sharp edge of the bumper of his_________’ the next target word is *avtomobil’a* ‘car’ which was correctly predicted by 94% of participants in the cloze task but obtained a 0.25 probability in the corpus. The other plausible continuations that the LSTM model proposed were synonyms of the word ‘car’, such as ‘SUV’ and ‘jeep’. In most of the other cases, the LSTM model suggested the target word as the second or the third plausible word for highly constrained sentences like ‘Indian Ocean’ or ‘cozy home’.

#### 3.2.2. Morphosyntactic probabilities

The participants in the cloze experiment and the LSTM model both predicted word class information of upcoming words more accurately than words in their particular grammatical forms (lexical probabilities). Table 2 indicates that word class can be highly predictable from context: mean word class probabilities reach 60%, whereas lexical probabilities do not exceed 20%. This shows that the word class of an unpredictable word can still be highly predictable. Our result speaks in favor of syntactic prediction in Russian, and is in line with the study by Luke and Christianson [13] for English.

**Table 2:** Mean word class probabilities and standard deviations.

Word classes	# words	Mean word class cloze probabilities	Mean word class corpus-based probabilities	Pearson correlations
Content words				
nouns	439	0.76 (0.01)	0.81 (0.02)	0.71
verbs (finite forms)	190	0.66 (0.02)	0.70 (0.03)	0.63
verbs (infinitives)	52	0.65 (0.05)	0.71 (0.06)	0.72
adjectives	165	0.35 (0.02)	0.32 (0.04)	0.57
adverbs	44	0.30 (0.05)	0.16 (0.06)	0.72
numerals	6	0.45 (0.15)	0.50 (0.20)	0.00
**All content words**	896	0.63 (0.01)	0.66 (0.02)	0.70
**Function words**				
personal pronouns	69	0.47 (0.03)	0.36 (0.06)	0.67
prepositions	117	0.71 (0.03)	0.60 (0.05)	0.59
conjunctions	64	0.74 (0.03)	0.55 (0.06)	0.64
particles	32	0.52 (0.05)	0.50 (0.09)	0.71
**All function words**	282	0.63 (0.02)	0.52 (0.03)	0.65
**All words**	1178	0.63 (0.01)	0.62 (0.01)	0.68

To check whether high mean word class probability could be explained only by a small number of classes or skewed class distribution, we established a baseline model which assigned probabilities according to a unigram distribution, e.g. it assigned 37% probability for nouns, 14% for adjectives, etc. Mean word class probability in this baseline is only 20%, compared to 63% and 62% for cloze and corpus-based probabilities respectively.

We qualitatively analyzed 102 words for which word classes were correctly predicted by participants in the cloze task (i.e. the majority of words suggested by participants matched in word classes with the target words) but incorrectly predicted by the LSTM model (i.e. the majority of words suggested by the model did not match in word classes with the target words), and found that only 39 words reached a word class probability score of 0.5 in the cloze experiment. For most of these mismatch cases, variability in human answers as well as in LSTM predictions is high. In particular, the LSTM model usually incorrectly predicted nouns instead of target verbs, prepositions, and personal pronouns; however the correct tags for these word classes were among the top-five most probable tags suggested by the model. Nouns were mostly confused with adjectives; that is, the model predicted adjectives instead of target nouns while the participants in the cloze experiment correctly predicted nouns. We believe that the latter can be explained by the fact that the LSTM model was trained on written texts in which nouns are often preceded by adjectives.

Next, we looked into the predictability of morphological features for nouns and verbs when the target word was indeed a noun or a verb. For nouns, both the participants in the cloze task and the LSTM model predicted their number and case very accurately (Table 3). However, for noun gender (Russian has three genders), cloze probabilities were higher than corpus-based probabilities, indicating that people seem to be more sensitive to gender in context in comparison with the language model. For verbs, human participants were more accurate than the LSTM model in predicting gender, tense, and person markup (see Table 4). Overall, the mean morphological cloze and corpus-based probabilities are higher than the lexical probabilities. This suggests that morphological information can be predictable in context, even when the particular word is not predictable.

**Table 3:** Mean morphological probabilities for nouns and standard deviations.

	Mean morphological cloze probabilities	Mean morphological corpus-based probabilities	Pearson correlations
gender	0.62 (0.02)	0.51 (0.02)	0.69
number	0.86 (0.01)	0.83 (0.02)	0.64
case	0.86 (0.01)	0.81 (0.02)	0.58
**All features**	0.78 (0.01)	0.51 (0.01)	0.67

**Table 4:** Mean morphological probabilities for finite forms of verbs and standard deviations.

	Mean morphological cloze probabilities	Mean morphological corpus-based probabilities	Pearson correlations
tense	0.56 (0.02)	0.38 (0.04)	0.72
number	0.79 (0.02)	0.73 (0.03)	0.56
person	0.43 (0.04)	0.23 (0.06)	0.73
gender	0.66 (0.03)	0.53 (0.05)	0.71
**All features**	0.65 (0.01)	0.52 (0.02)	0.71

*Note*. Despite the fact that morphosyntactic probabilities were calculated based on lexical probabilities, they are qualitatively very different: a word that has low lexical probability can have high morphosyntactic probability if all the words suggested by the participants or the model share the same morphosyntactic features, and vice versa.

In summary, we found that mean cloze and corpus-based *lexical* probabilities were strongly correlated. The LSTM model systematically assigned lower probabilities to the words that were very probable in the cloze task, which led to a slightly lower correlation. Our results also indicate that *morphosyntactic* features of words are highly predictable in context, even when the particular lemma itself is not predictable. This conclusion is true for both cloze and corpus-based probabilities, since the two types of probabilities strongly correlate. However, there is some indication that the participants in the cloze task are more sensitive to the morphological features of nouns and verbs than the LSTM model.

## 4. Eyetracking experiment

### 4.1. Method

#### 4.1.1. Dataset

We used eye movements data originally reported by Laurinavichyute et al. [44] and freely available from DOI 10.17605/OSF.IO/X5Q2R. The dataset comprised data from ninety-six monolingual native speakers of Russian (66 female; mean age = 24, range 18–80 years, none participated in the cloze experiment) reading the same 144 sentences used in the close task. The first and last words in every sentence were excluded from the analyses: the first words were excluded because the initial fixation point forced an unnatural first fixation position on the word, and the last words were excluded to eliminate end-of-sentence wrapping effects. Overall, data on reading 1,074 words were included in the analyses of eye movements.

#### 4.1.2. Analyses

We assessed which of the two probability measures provided a better account for the observed word processing costs in the eyetracking experiment. To study *lexical predictability*, we fitted separate linear mixed-effects models for four commonly reported eye fixation measures as dependent variables: single fixation duration (SFD), first fixation duration (FFD), gaze duration (GD), and total reading time (TT). For each dependent measure, two models were created: one including cloze and the other including corpus-based probabilities as predictors. These sixteen pairs of models fitted for the same dependent variable were compared for goodness of fit using k-fold cross-validation.

In line with the previously reported analysis [44], each model included the following predictors: for the previous, currently fixated, and upcoming words, these were the centered and scaled word form length, logarithm (base 10) of word form frequency, and logit-transformed probabilities (either cloze or corpus-based); in addition, for the currently fixated words, the model included the amplitude of the incoming saccade, the landing position of the saccade, and the predictor distinguishing whether the word was in its base (dictionary) form or not. The models also included varying intercepts for participants, sentences, and individual words.

The models were fitted using the ‘brms’ package [47] for R [48], and the graphs were plotted using the ‘ggplot2’ and ‘tidybayes’ packages [49, 50]. The models were fitted using a Bayesian modeling approach with weakly informative priors (see priors listed in Supporting information, S1 Table). All models assumed lognormal distribution of the dependent variable.

To find out whether eye movements are influenced not only by lexical predictability, but additionally by *morphosyntactic predictability*, we extended the models described above (see the overview in Table 5). First, we added independent variables encoding the word class probability (either cloze or corpus-based) of the current and upcoming words. We included the word class probability of the upcoming word since it might affect parafoveal processing of this word and, therefore, the duration of fixations on the current word. We did not include the word class probability of the previous word because by the time of processing the current word the information is already available, irrespective of whether or not it was predicted correctly. Second, on top of the models containing the word class probability information, we added probabilities (either cloze or corpus-based) of particular morphological features of the currently fixated word. We coded morphological probabilities only for nouns and verbs, and since the morphological features in question differ, we fitted separate models for nouns and verbs. For nouns, the added predictors were the probabilities of gender, case, and number marking. For verbs, the added predictors were the probabilities of tense, gender, number, and person. We fitted separate models for verbs in present/future and past tense forms, since in Russian’s conjugation system, in the present and future tenses, verbs mark person and number, but not gender; while in past tense forms, verbs mark number and gender, but not person.

**Table 5:** A summary of specifications of all fitted models.

Predictors shared across all models	n-1, n, n+1 word length,		
	n-1, n, n+1 word frequency,		
	amplitude of incoming saccade,		
	saccade landing position,		
	base/non-base word form		
+ Baseline model	Cloze: n-1, n, n+1 word probability		
	Corpus: n-1, n, n+1 word probability		
+ Word class predictability	Cloze: n, n+1 word class probability		
	Corpus: n, n+1 word class probability		
	+ Grammatical characteristics of nouns	+ Grammatical characteristics of verbs (present and future tense)	+ Grammatical characteristics of verbs (past tense)
	Cloze: gender, case, and number marking probability	Cloze: tense, person and number marking probability	Cloze: tense, gender and number marking probability
	Corpus: gender, case, and number marking probability	Corpus: tense, person and number marking probability	Corpus: tense, gender and number marking probability

*Note*. Composition of fitted models: each model included predictors described in its own cell and predictors from the models above. That is, the model for word class predictability included predictors of word class probability (either cloze or corpus-based), predictors from the baseline model, and the predictors shared across all models.

We compared the goodness of fit of the models with cloze and corpus-based probabilities to find out which of the models explained more variance in eye fixation durations. The comparison was done using the k-fold information criterion in the ‘brms’ package with k equal to 10, due to leave-one-out cross-validation being computationally infeasible. The difference between the k-fold information criteria of goodness of fit for the models with cloze and corpus-based probabilities (cloze minus corpus) will show which of the two probabilities is a better predictor of variance in eye movements while reading. Overall, we will make twenty comparisons: four for lexical probabilities and sixteen for morphosyntactic probabilities. If models containing cloze probabilities perform on par with the models containing corpus-based probabilities, then both probability measures explain the same amount of variance.

Even if we find that cloze and corpus-based measures explain the same amount of variance, we still cannot conclude that the measures are identical. Cloze probabilities might better explain the variance associated with high- and medium-predictable words, while corpus-based probabilities might better account for the processing of low-predictable words. To assess whether cloze and corpus measures indeed account for the same variance in eye movements, we need to test whether cloze probabilities explain any variance on top of what is explained by corpus-based probabilities, and vice versa. A straightforward way to do that would be to add both measures into the same model as predictors, but we cannot do that due to high correlation between the measures. However, we can extract the residuals of each model we fit (residuals representing the variance that the predictors cannot account for) and use these residuals as a dependent variable for a model that would include complementary measures of predictability: if the original model included human cloze probabilities, then we regress corpus-based probabilities on the model’s residuals, and vice versa.

### 4.2. Results

To explore the impact of cloze and corpus-based probabilities on eye movements while reading, we conducted a series of analyses of the eyetracking data. The dependent variables included single fixation duration, first fixation duration, gaze duration, and total reading time. The overview of all independent variables is presented in Table 5.

**Fig 3:**
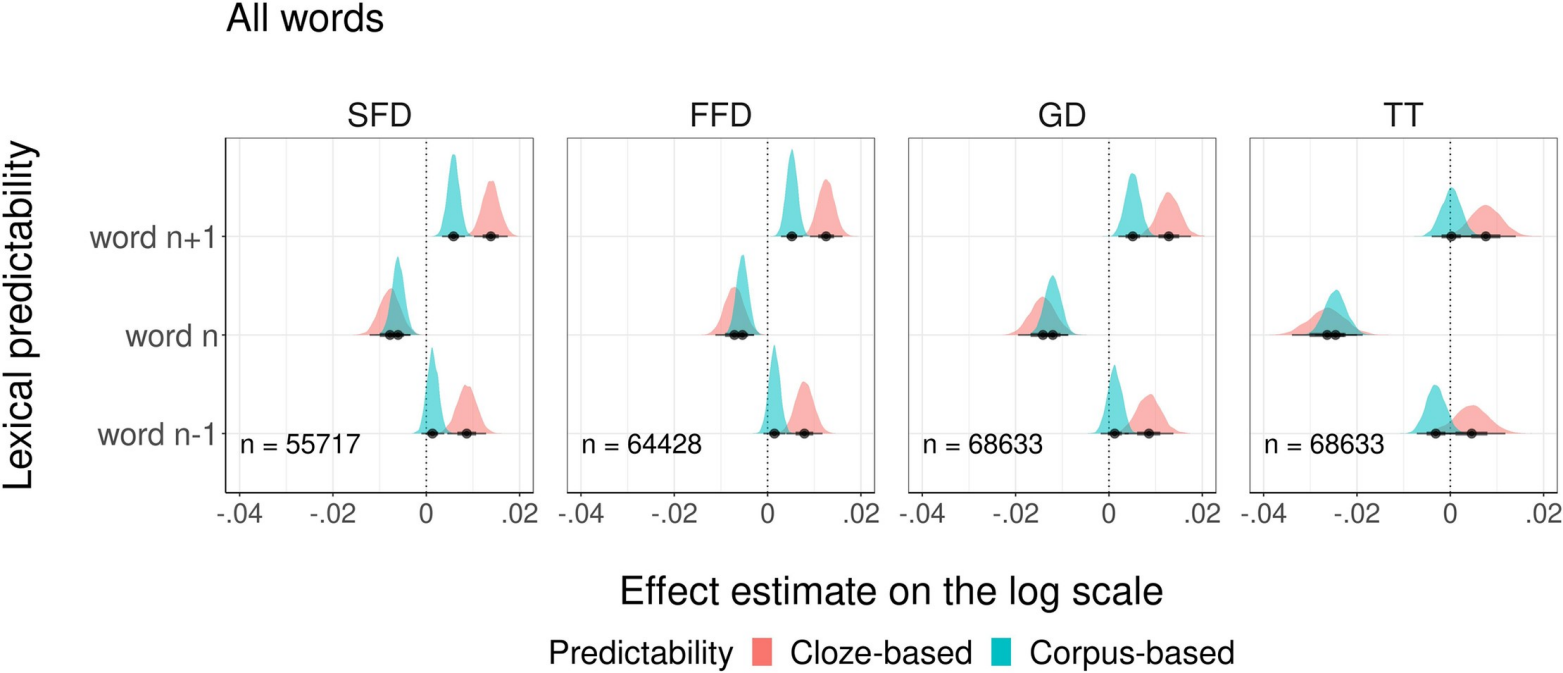
The impact of cloze and corpus-based lexical probabilities for the currently fixated, previous, and next words on four fixation duration measures.

First, we compared cloze and corpus-based *lexical probabilities*, referring to the probabilities of encountering a particular word in a particular grammatical form in context. The graphical overview of modeling results is presented in Fig 3. We plotted the estimated impact of cloze and corpus-based probabilities of the currently fixated word, the previous and the next words on each of the four dependent measures. Detailed summaries of model fits can be found in the Supporting information (S2 Table). To briefly review the outcomes, an increase in the probability (both cloze and corpus-based) of the currently fixated word reliably decreased SFD, FFD, GD, and TT on that word. For the upcoming words, their greater cloze and corpus-based probabilities reliably increased SFD, FFD, GD, and TT (only cloze probabilities for this measure) on the currently fixated word. Higher cloze but not corpus-based probabilities of the previous word increased SFD, FFD, and GD on the currently fixated word. Neither cloze nor corpus-based probabilities of the previous word affected TT on the current word.

We also compared the goodness of fit to the eye movement data for each pair of models. We found that for all dependent measures, models with cloze and corpus-based probabilities did not differ in the goodness of fit (see Table 6). These results indicate that the two types of probability explain comparable amounts of variance in eye movements while reading.

**Table 6:** The difference between k-fold information criteria of the goodness of fit for the models with cloze and corpus-based lexical probabilities.

	k-fold IC	SE	k-fold IC / SE
SFD (cloze–corpus)	-6.91	25.98	-0.27
FFD (cloze–corpus)	3.22	11.66	0.28
GD (cloze–corpus)	-23.6	27.16	-0.87
TT (cloze–corpus)	-29.03	27.43	-1.06

*Note*. We subtracted k-fold information criterion for the model with corpus-based lexical probabilities from k-fold information criterion for the model with cloze lexical probabilities. Negative numbers indicate that models with cloze lexical probabilities have a better goodness of fit than models with corpus-based lexical probabilities and vice versa.

Analysis of residuals showed that although cloze and corpus-based probabilities explain similar amount of variance in the data, they do not explain the same variance (see summary Table 7 and S2 Table for more details). For each model, at least one out of three complementary probability measures was a reliable predictor of variation in residuals (if the original model included cloze probabilities, then the model built on residuals from that original model included three corpus-based probabilities—that of the previous word, the currently fixated word, and of the upcoming word).

**Table 7:** The summary of the analysis of residuals for lexical and morphosyntactic probabilities.

	SFD (corpus on cloze)		SFD (cloze on corpus)		FFD (corpus on cloze)		FFD (cloze on corpus)		GD (corpus on cloze)		GD (cloze on corpus)		TT (corpus on cloze)		TT (cloze on corpus)	
*Predictors*	*Estimates*	*HDI (95%)*	*Estimates*	*HDI (95%)*	*Estimates*	*HDI (95%)*	*Estimates*	*HDI (95%)*	*Estimates*	*HDI (95%)*	*Estimates*	*HDI (95%)*	*Estimates*	*HDI (95%)*	*Estimates*	*HDI (95%)*
Intercept	-0.98	-2.48–0.48	0.98	-0.30–2.25	-1.00	-2.43–0.40	0.78	-0.43–1.95	-4.41	-6.47 –-2.07	-0.50	-2.46–1.51	-10.05	-13.90– -6.17	-3.21	-6.57–0.17
n lexical probability	**-0.40**	**-0.69 –-0.11**			**-0.39**	**-0.68 –-0.12**			**-2.33**	**-2.78 –-1.88**	**-3.16**	**-4.02 –-2.28**	**-4.28**	**-5.03 –-3.52**	**-6.12**	**-7.61 –-4.66**
n+1 lexical probability							**0.53**	**0.08–0.96**					**-0.95**	**-1.68 –-0.23**	**-1.27**	**-2.52 –-0.08**
n-1 lexical probability											**0.98**	**0.12–1.86**				
Intercept									-4.79	-7.26 –-2.37						
n word class probability									**1.24**	**0.07–2.40**						
Intercept											-8.57	-22.63–5.45			-13.92	-36.76–8.37
n tense probability (for present and future)											**-6.56 **	**-12.24 –-0.75**			**-10.01**	**-19.32 –-0.96**
															

*Note*. In this summary table, we highlight only predictors that explained non-overlapping variance in the models with lexical probabilities and the models with word class probabilities and morphological probabilities for verbs in the present and future tenses. The outputs of the models with morphological probabilities for nouns and for verbs in the past tense are not presented because morphological predictors in them do not explain non-overlapping variance. The table can be read as follows: for SFD, the corpus-based lexical probability of word n explains variance over and above the cloze-based probability of word n (see column SFD (corpus on cloze)). However, the cloze-based lexical probability of word n does not explain any variance beyond that accounted for by the corpus-based probability of word n (see column SFD (cloze on corpus)). For more details, see S2–S6 Tables.

**Fig 4:**
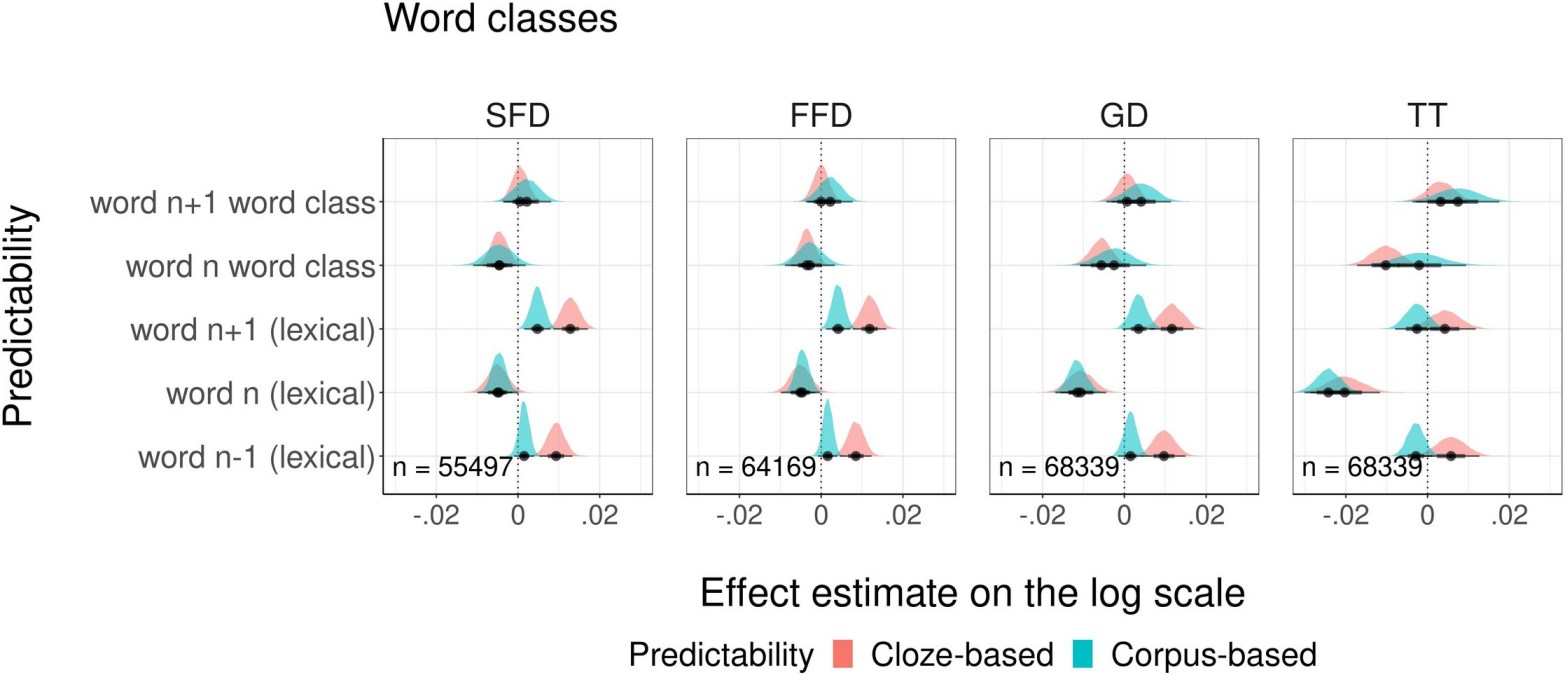
Visualization of modeling estimates for four fixation duration measures.

Next, we studied whether partial predictions about the morphosyntactic features of upcoming words affect reading times. For that purpose, we controlled for lexical predictability and entered the probabilities of different morphosyntactic features, along with lexical probabilities, into the same models. As before, we compared probabilities from the cloze task with corpus-based probabilities from the LSTM model. The first comparison included models with cloze and corpus-based *word class probabilities*. The graphical overview of modeled estimates is presented in Fig 4. We plotted the impact of cloze and corpus-based word class probabilities of the currently fixated word and the next words on the four dependent measures. Detailed summaries of model fits are presented in the Supporting information (S3 Table). We found that higher cloze word class probabilities of the currently fixated word (but not the next word) decreased SFD, GD, and TT. These findings suggest that word class probabilities facilitate reading. Corpus-based probabilities did not affect reading speed. But the comparison of the goodness of fit for each pair of models showed a significant difference in goodness of fit only for GD: the model with cloze probabilities explained more variance in eye movements than the model with corpus-based probabilities (see Table 8).

**Table 8:** The difference between k-fold information criteria of the goodness of fit for the models with cloze and corpus-based word class and morphosyntactic probabilities.

**Cloze and corpus-based word class probabilities**			
	**k-fold IC**	**SE**	**k-fold IC / SE**
SFD (cloze–corpus)	-44.51	25.83	-1.72
FFD (cloze–corpus)	-5.57	12.85	-0.43
GD (cloze–corpus)	**-65.89**	**27.33**	**-2.41**
TT (cloze–corpus)	29.65	26.92	1.10
**Cloze and corpus-based morphological probabilities for nouns**			
SFD (cloze–corpus)	29.45	17.61	1.67
FFD (cloze–corpus)	7.48	10.24	0.73
GD (cloze–corpus)	-7.36	17.66	-0.42
TT (cloze–corpus)	10.14	18.44	0.55
**Cloze and corpus-based morphological probabilities for verbs in present and future tenses**			
SFD (cloze–corpus)	17.46	11.97	1.46
FFD (cloze–corpus)	5.58	7.46	0.75
GD (cloze–corpus)	-5.98	11.83	-0.51
TT (cloze–corpus)	6.75	11.63	0.58
**Cloze and corpus-based morphological probabilities for verbs in the past tense**			
SFD (cloze–corpus)	-15.83	12.76	-1.24
FFD (cloze–corpus)	-0.27	7.74	-0.03
GD (cloze–corpus)	-3.76	13.51	-0.28
TT (cloze–corpus)	**-31.15**	**13.42**	**-2.32**

*Note*. Negative numbers indicate that models with cloze probabilities have a better goodness of fit than models with corpus-based probabilities and vice versa. Significant results are in bold.

Analysis of residuals again showed that although cloze and corpus-based probabilities explain similar amounts of variance in most of the measures, they do not explain the same variance (see summary Table 7 and S3 Table for more details). For each model, at least one out of five complementary probability measures was a reliable predictor of variation in residuals, for GD and TT measures two out of five probability measures were reliable predictors. However, only one of these measures in one model was related to word class probabilities (for GD, corpus-based word class probability of the current word was a reliable predictor in the model built on residuals from cloze-based probabilities), the rest of non-overlapping variability in the eye-movement data was explained by lexical probabilities of the current word estimated from cloze and corpus.

**Fig 5:**
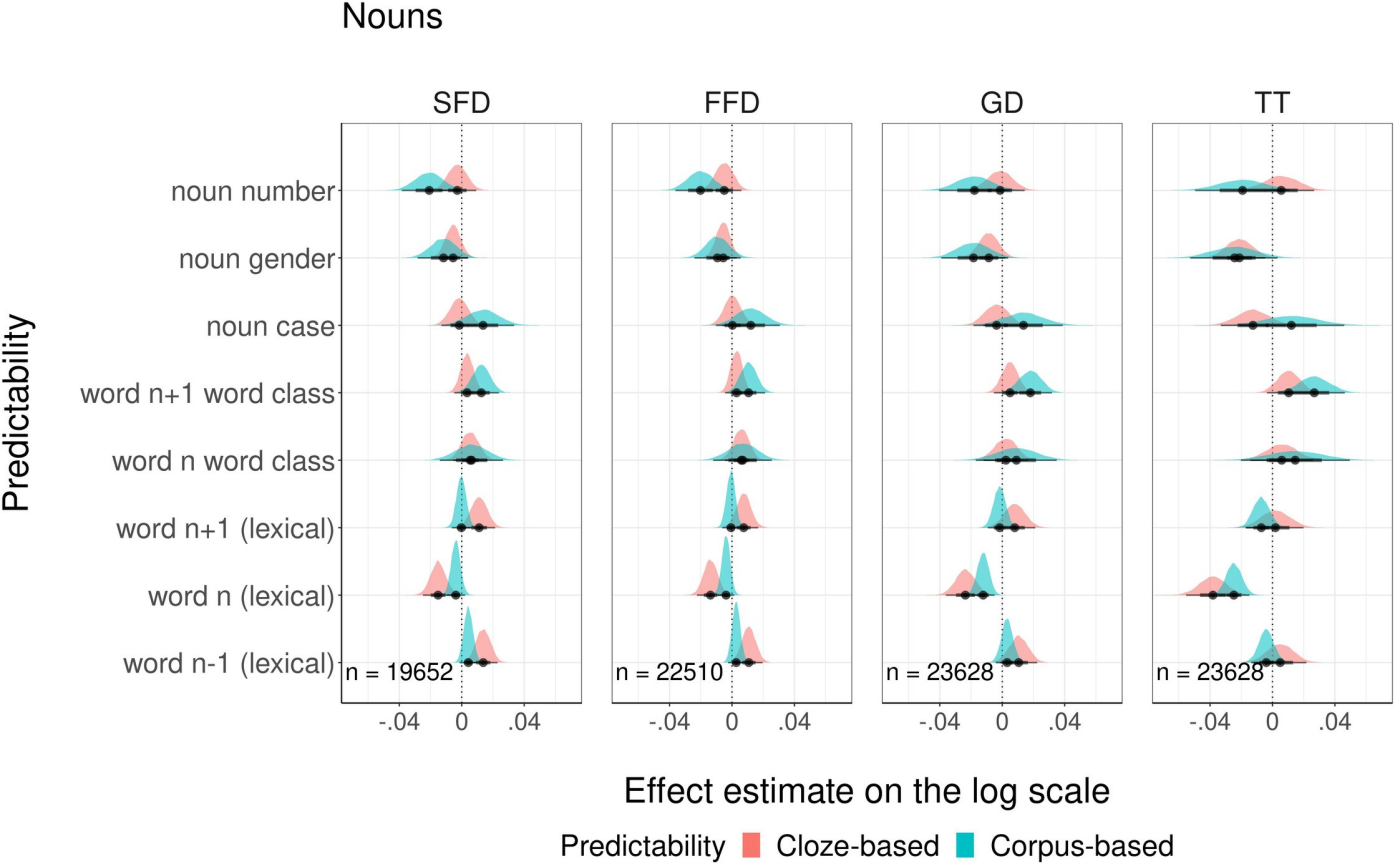
Visualization of modeling estimates for four fixation duration measures.

The second comparison included models with cloze and corpus-based *morphological probabilities for nouns*. A graphical overview of the modeled estimates is presented in Fig 5. Detailed summaries of model fits are presented in the Supporting information (S4 Table). We found that higher cloze probabilities of gender decreased TT and that higher corpus-based probabilities of number marking decreased SFD and FFD. There is no evidence that other morphological probability predictors affected reading times. For all dependent variables, there was no significant difference in goodness of fit to the eye movement data between models with cloze and corpus-based probabilities (see Table 8). Analysis of model residuals showed that for SFD and FFD models, none of eight complementary probability measures predicted variation in the residuals, whereas for GD and TT measures, one out of eight measures was a reliable predictor of variation in the residuals (see S4 Table). However, this complimentary predictor was always the lexical predictability of the current word, and not any measure of morphological probabilities of noun features.

**Fig 6:**
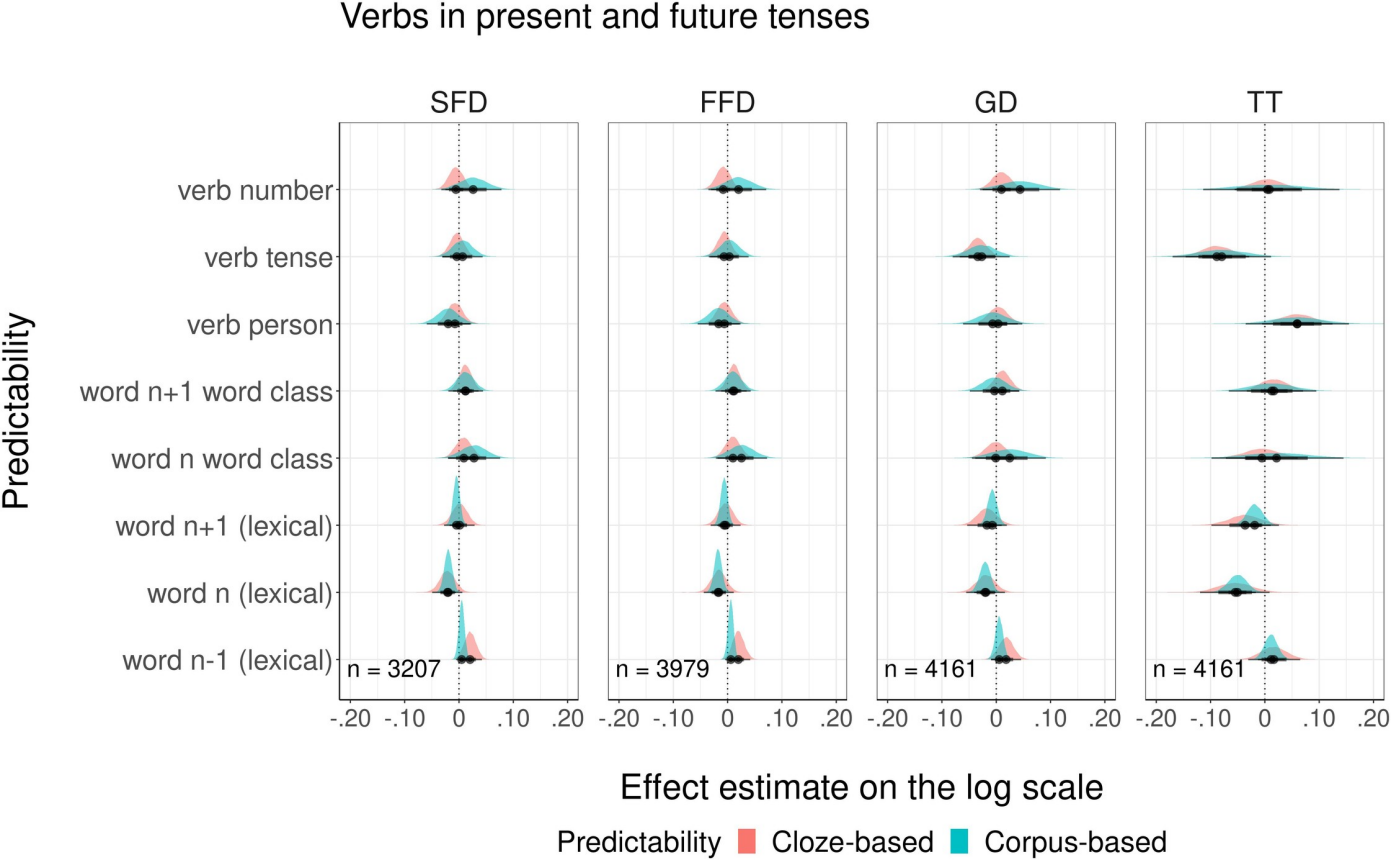
Visualization of modeling estimates for four fixation duration measures.

The third comparison focused on models with cloze and corpus-based *morphological probabilities for verbs in the present and future tenses*. The graphical overview of modeled estimates is presented in Fig 6. Detailed summaries of model fits are presented in the Supporting information (S5 Table). The results showed that cloze but not corpus-based higher probabilities of tense decreased GD and TT. There was no evidence for any influence of other morphological probability predictors. For all dependent variables, there was no significant difference in goodness of fit to the eye movement data between models with cloze and corpus-based probabilities (see Table 8). Analysis of model residuals showed that for SFD and FFD models, none of eight complementary probability measures predicted variation in the residuals, whereas for GD and TT measures, one out of eight measures was a reliable predictor (see summary Table 7 and S5 Table for more details). For the models using residuals from cloze probabilities, the corpus-based lexical probability of the current word was this predictor, while for the models using residuals from corpus probabilities, the influential predictor was the cloze probability of tense marking.

**Fig 7:**
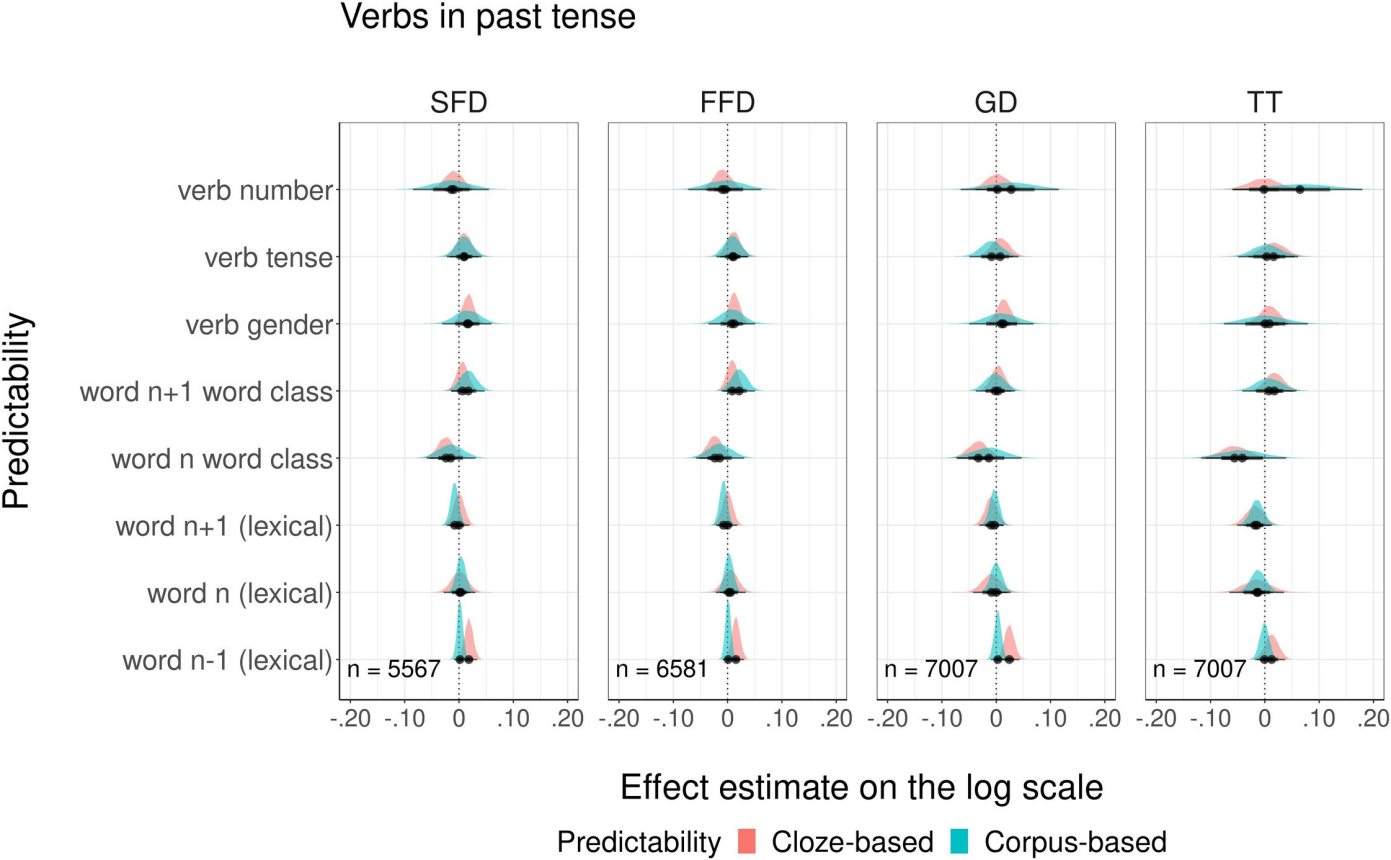
Visualization of modeling estimates for four fixation duration measures.

The last comparison included models with cloze and corpus-based *morphological probabilities for verbs in the past tense*. The graphical overview of modeled estimates is presented in Fig 7. Detailed summaries of model fits are presented in the Supporting information (S6 Table). The results showed that neither cloze nor corpus-based morphological probabilities affected reading times. Nevertheless, there was a significant difference in goodness of fit between TT models: the model with cloze probabilities explained more variance in eye movements than the model with corpus-based probabilities (see Table 8). Analysis of model residuals showed that for SFD and FFD models, none of eight complementary probability measures predicted variation in the residuals, whereas for GD and TT measures, one out of eight measures was a reliable predictor (see S6 Table). However, none of these measures was related to morphological probabilities, the non-overlapping variability in the eye-movement data was explained by lexical probabilities of the currently fixated word estimated from cloze and corpus.

To summarize, our results suggest that cloze and corpus-based *lexical* probabilities explain comparable amounts of variance in eye fixation durations while reading: we did not find any difference in goodness of fit for the SFD, GD, and TT models. However, the analysis of model residuals showed a consistent pattern of cloze probabilities explaining significant variance in the residuals of models that included corpus-based probabilities, and vice versa. Since cloze and corpus-based probabilities explain non-overlapping variability in the eye-movement data, we conclude that these measures cannot be considered to be sufficiently equivalent. The comparison of the goodness of fit for the models with either cloze or corpus-based *morphosyntactic* probabilities did not reveal any difference in fourteen out of sixteen models. We found better goodness of fit for the GD and TT models with cloze morphosyntactic probabilities, but two cases out of sixteen in different models and different measures cannot be considered as systematic and might result from random fluctuations. We suggest that these results do not constitute strong evidence in favor of cloze probabilities. Similarly, while we observed single instances of cloze morphosyntactic probabilities being important predictors of variance on top of corpus-based probabilities, and vice versa, these effects were by no means systematic. The only systematic contribution came from lexical probabilities: both cloze and corpus-based lexical probabilities systematically explained non-overlapping variance in eye movements while reading.

## 5. Discussion

The purpose of the present study was to compare human-based and corpus-based approaches to measuring predictability. We selected the best predictability model, obtained four types of probabilities (cloze and corpus-based lexical and morphosyntactic probabilities), correlated them in pairs, and included each of them as a predictor of variance in an eyetracking-while-reading experiment. First, we confirmed that the LSTM predictability model outperformed the 5-gram model: the former was more accurate and estimated probability better than the latter, which agrees with the work of Jozefowicz and colleagues [41]. Our main finding is that corpus-based lexical probabilities cannot completely substitute for cloze in reading experiments. Specifically, we showed that although cloze and corpus-based lexical probabilities were strongly correlated and explained comparable amounts of variance in an eyetracking experiment, they accounted for different sources of variance. Cloze and corpus-based morphosyntactic probabilities were also strongly correlated and, unlike lexical probabilities, explained variance of the same source.

We found that cloze and corpus-based *lexical probabilities* strongly correlate for low predictability words. For highly predictable words, correlation is slightly lower, and overall corpus-based probabilities underestimate cloze probabilities, even though their rank may be the same. This skewness can be explained by the nature of cloze and corpus-based probabilities. Cloze probabilities estimate the distribution of word probabilities within the mind of a participant indirectly, see [18, 25]. In the cloze task, we assume that each participant produces answers by sampling from her probability distribution and provides a single most probable word. Taken together, these words should reflect the distribution of word probabilities that the participants have in mind. But for corpus-based probabilities, we directly deal with the distribution of probabilities: when the language model predicts a word, it immediately produces probabilities for all words in its vocabulary. Therefore, even in constraining contexts in which all participants of the cloze task might provide the same word, the model might assign some probability to less probable but also plausible words from its vocabulary. If we look for conditional probability estimates that directly reflect probability distributions, we should opt for corpus-based probabilities instead of cloze probabilities.

The eyetracking experiment showed that an increase in both cloze and corpus-based lexical probabilities decreases single fixation duration (SFD), first fixation duration (FFD), gaze duration (GD), and total reading time (TT) for the currently fixated words—more predictable words required shorter fixation durations. Interestingly, for the previous words, whose probabilities were also included as predictors of fixation duration on the current word, there was a small but systematic difference between cloze and corpus-based measures. Cloze but not corpus-based probabilities of the previous word increased SFD, FFD, and GD on the currently fixated word. However, a comparison of the goodness of fit for the models with either cloze or corpus-based probabilities did not reveal any significant difference between the models, and thus, the two types of probabilities explain comparable amounts of variance in eye movements while reading. Importantly, we showed that the source of variance explained by cloze probabilities differed from the source of variance explained by corpus-based probabilities. We suppose that cloze probabilities better explain the variance associated with highly predictable words whereas corpus-based probabilities better accounted for less predictable words. Overall, although both direct correlation measures and the results of the eyetracking experiment indicate that corpus-based lexical probabilities are comparable with cloze probabilities, we cannot conclude that the former could substitute for the latter, because the two measures account for different sources of variance in word processing while reading.

As we expected, participants successfully predicted the morphosyntactic features of Russian words, and this *morphosyntactic predictability* went beyond lexical predictability. Particularly, both the participants in the cloze task and the LSTM model successfully predicted the word class of upcoming words as well as particular morphological features of upcoming nouns and verbs (most likely, of other word classes as well, but we only tested nouns and verbs). In the cloze task, the average accuracy of morphosyntactic prediction was as high as 70% as compared to lexical prediction which reached only 18%. In the eyetracking data, predictable word class, number and gender in nouns, and present/future tense in verbs speed up reading even when we controlled for lexical predictability and all variables traditionally controlled for in reading experiments (see Section 4.1.4). These results confirm and expand the findings of Luke and Christianson [13] for English, and suggest that people indeed engage in partial morphosyntactic prediction that facilitates the processing of upcoming input. Moreover, our data suggest that people generate predictions about all existing morphosyntactic features of upcoming words much more often or at least more successfully than about their full identities. It follows that in languages with rich inflectional morphology, prediction implies not only and definitely not exclusively pre-activation of particular word forms, but rather pre-activation of the morphosyntactic features of upcoming words. Further research is needed that explores other morphosyntactic features in Russian, as well as in other morphologically rich languages, to explore the limits and the nature of prediction.

However, not all predictable morphological features facilitate reading. Surprisingly, noun case probability as well as verb number, person, gender, and past tense probabilities, which the participants of the cloze task and the LSTM model predicted with high precision, showed no facilitative effect in reading. One possible explanation is that, overall, predictability does not account for large amounts of variance in eye movements. Therefore, the lack of evidence for any facilitative effect might be due to the tiny effect size that the models cannot capture. Note that for nouns we had about 23,000 observations whereas for verbs the maximum number of observations reached only 7,007. Another explanation is the possible asymmetry in predictability cues between production and comprehension. For example, case was highly predictable in the cloze task in our study, but Russian-speaking readers tend to ignore it when processing syntactically unambiguous sentences of a particular structure [51, 52]. The agreement features of Russian nouns and verbs might be relatively unimportant in comprehension, since readers might rely on other cues such as word class information or lexical semantics when building sentence representations.

Not only the participants of the cloze task, but also the LSTM model successfully predicted morphosyntactic probabilities: direct comparison of cloze and corpus-based morphosyntactic probabilities showed a strong correlation (R = 0.68) for all word classes. The two measures diverge in low predictability forms occurring in unconstrained contexts that were hard for both the human participants and the LSTM model. The predicted morphological features of nouns and verbs also showed a strong correlation: 0.67 for nouns and 0.71 for verbs. But morphological probabilities from the cloze task are higher than those from the LSTM model, indicating that human participants are more sensitive to the morphological features of upcoming nouns and verbs than the language model. The results of the eyetracking experiment show that cloze and corpus-based probabilities predict a comparable amount of variance in eye fixation durations. Importantly, our results indicate that both measures accounted for variance from the same source. Still, there might be some indication that the morphosyntactic probabilities obtained in the cloze task explain more variance in fixation durations while reading: the two models (out of sixteen) with cloze probabilities had better goodness of fit than the models with corpus-based probabilities. Possibly, even a small gain in probabilities that the participants provided for our experimental words in the cloze task could have led to a greater amount of explained variance as compared to corpus-based probabilities. Overall, the difference in explained variance was negligible, allowing us to conclude that corpus-based morphosyntactic probabilities can be successfully used as predictors of variance in eyetracking experiments that study morphosyntactic prediction instead of cloze probabilities.

To close the gap between cloze and corpus-based probabilities, one could select a better training corpus for language models. We compared the performance of the models trained on three text corpora of Russian: the state-of-the-art Russian National Corpus (RNC), the recently released Taiga corpus that contained more contemporary examples of the written language, and the combination of RNC+Taiga. We found that the LSTM model trained only on RNC showed the best results on the test sentences, even though RNC was smaller than RNC+Taiga (577 million tokens versus 1,077 million tokens). This indicates that the content similarity of the training corpus to the test corpus is more important than the corpus size. Ultimately, for comprehension experiments, it means that the content similarity of a model’s training corpus to experimental stimuli sentences should influence the similarity of probability distributions from the language model to probability distributions that the participants of an experiment have in their minds when they read the stimuli sentences. For example, a model trained on a corpus of fiction will be a bad estimate of probabilities for oral speech samples. Brysbaert, Buchmeier, Conrad, Jacobs, Bölte, and Böhl [53] came to a similar conclusion for word frequency measures: frequencies obtained from a colloquial corpus of subtitles predicted word recognition speed better than frequencies from written language corpora. Therefore, the corpus-based probabilities from our study could be improved by selecting a model training corpus with more colloquial language.

Generally, our data suggest that corpus-based lexical and morphosyntactic probabilities show very similar results to cloze probabilities. As the difference between cloze and corpus-based morphosyntactic probabilities as predictors of reading times was tiny and nonsystematic, we conclude that corpus can substitute for cloze in estimating morphosyntactic predictability in reading studies. However, cloze and corpus-based lexical probabilities cannot be considered equivalent: the two measures explain variance in eye fixation durations that comes from different sources. If cloze lexical probability is indeed a measure of relative activation of an item in context [25], and corpus-based lexical probability is a conditional probability of an item, then the two types of probabilities capture different cognitive processes and should account for slightly different variance in reading behavior. Therefore, by having access to both types of lexical predictability estimates, we should decide for each particular study what exactly we want to study or control for—cloze probabilities (that account for highly predictable words and overlook low predictability words) or corpus-based conditional probabilities (that account for less predictable words but are not precise estimates for highly predictable words)—and what predictability we want to estimate.

## Supporting information

S1 Table Weakly informative priors for the linear models. (PDF)

S2 Table Summaries of model fits with either cloze or corpus-based lexical probabilities. (PDF)

S3 Table Summaries of model fits with either cloze or corpus-based word class probabilities. (PDF)

S4 Table Summaries of model fits with either cloze or corpus-based morphological probabilities for nouns. (PDF)

S5 Table Summaries of model fits with either cloze or corpus-based morphological probabilities for verbs in present and future tenses. (PDF)

S6 Table Summaries of model fits with either cloze or corpus-based morphological probabilities for verbs in past tense. (PDF)
