# Supplementary material for: Morphosyntactic but not lexical corpus-based probabilities can substitute for cloze probabilities in reading experiments
Source: PLoS One. 2021 Jan 28;16(1):e0246133. doi: 10.1371/journal.pone.0246133 (PMC7842903; doi:10.1371/journal.pone.0246133)
Supplement: S1 Table — (PDF) [file pone.0246133.s001.pdf]

S1 Table. Weakly informative priors for the linear models.

Measure	Priors (on the log scale)
SFD	Intercept — normal(5.4, 0.5)<br>beta — normal(0, 0.1)<br>sd — normal(0, 0.1)<br>sigma — normal(0, 0.7)
FFD	Intercept — normal(5.3, 0.4)<br>beta — normal(0, 0.1)<br>sd — normal(0, 0.1)<br>sigma — normal(0, 0.7)
GD	Intercept — normal(5.5, 0.7)<br>beta — normal(0, 0.5)<br>sd — normal(0, 0.3)<br>sigma — normal(0, 1)
TT	Intercept — normal(5.7, 0.7)<br>beta — normal(0, 0.5)<br>sd — normal(0, 0.3)<br>sigma — normal(0, 1)
