# Supplementary material for: Morphosyntactic but not lexical corpus-based probabilities can substitute for cloze probabilities in reading experiments
Source: PLoS One. 2021 Jan 28;16(1):e0246133. doi: 10.1371/journal.pone.0246133 (PMC7842903; doi:10.1371/journal.pone.0246133)
Supplement: S2 Table — (PDF) [file pone.0246133.s002.pdf]

S2 Table. Summaries of model fits with either cloze or corpus-based lexical probabilities.

	SFD (cloze probability)		SFD (corpus probability)		FFD (cloze probability)		FFD (corpus probability)		GD (cloze probability)		GD (corpus probability)		TT (cloze probability)		TT (corpus probability)	
Predictors	*Estimates*	*HDI (95%)*	*Estimates*	*HDI (95%)*	*Estimates*	*HDI (95%)*	*Estimates*	*HDI (95%)*	*Estimates*	*HDI (95%)*	*Estimates*	*HDI (95%)*	*Estimates*	*HDI (95%)*	*Estimates*	*HDI (95%)*
Intercept	5.42	5.39 – 5.45	5.40	5.36 – 5.43	5.41	5.38 – 5.44	5.39	5.35 – 5.42	5.54	5.50 – 5.58	5.50	5.45 – 5.54	5.69	5.63 – 5.74	5.59	5.53 – 5.66
frequency	-0.02	-0.03 – -0.02	-0.02	-0.03 – -0.01	-0.02	-0.03 – -0.02	-0.02	-0.02 – -0.01	-0.03	-0.04 – -0.03	-0.03	-0.03 – -0.02	-0.04	-0.05 – -0.04	-0.03	-0.04 – -0.02
n lexical probability	**-0.01**	**-0.01 – -0.00**	**-0.01**	**-0.01 – -0.00**	**-0.01**	**-0.01 – -0.00**	**-0.01**	**-0.01 – -0.00**	**-0.01**	**-0.02 – -0.01**	**-0.01**	**-0.02 – -0.01**	**-0.03**	**-0.03 – -0.02**	**-0.02**	**-0.03 – -0.02**
length	0.00	-0.00 – 0.00	0.00	-0.00 – 0.00	-0.00	-0.00 – 0.00	-0.00	-0.00 – 0.00	0.02	0.02 – 0.02	0.02	0.02 – 0.02	0.03	0.02 – 0.03	0.03	0.02 – 0.03
n+1 length	-0.03	-0.03 – -0.02	-0.03	-0.03 – -0.02	-0.02	-0.03 – -0.02	-0.02	-0.03 – -0.02	-0.03	-0.04 – -0.03	-0.03	-0.04 – -0.03	-0.04	-0.05 – -0.03	-0.04	-0.05 – -0.03
n+1 frequency	-0.02	-0.02 – -0.01	-0.02	-0.02 – -0.01	-0.01	-0.02 – -0.01	-0.01	-0.02 – -0.01	-0.02	-0.03 – -0.01	-0.02	-0.02 – -0.01	-0.03	-0.03 – -0.02	-0.02	-0.03 – -0.01
n+1 lexical probability	**0.01**	**0.01 – 0.02**	**0.01**	**0.00 – 0.01**	**0.01**	**0.01 – 0.02**	**0.01**	**0.00 – 0.01**	**0.01**	**0.01 – 0.02**	**0.01**	**0.00 – 0.01**	**0.01**	**0.00 – 0.01**	0.00	-0.00 – 0.00
n-1 length	0.00	-0.00 – 0.01	0.00	-0.00 – 0.01	0.00	-0.00 – 0.01	0.00	-0.00 – 0.01	-0.00	-0.01 – 0.01	-0.00	-0.01 – 0.01	-0.00	-0.02 – 0.01	-0.01	-0.02 – 0.00
n-1 frequency	-0.01	-0.01 – -0.00	-0.00	-0.01 – -0.00	-0.00	-0.01 – -0.00	-0.00	-0.01 – 0.00	-0.00	-0.01 – 0.00	-0.00	-0.01 – 0.00	-0.00	-0.01 – 0.01	0.00	-0.01 – 0.01
n-1 lexical probability	**0.01**	**0.00 – 0.01**	0.00	-0.00 – 0.00	**0.01**	**0.00 – 0.01**	0.00	-0.00 – 0.00	**0.01**	**0.00 – 0.01**	0.00	-0.00 – 0.00	0.00	-0.00 – 0.01	-0.00	-0.01 – 0.00
landing position	-0.01	-0.02 – -0.01	-0.01	-0.02 – -0.01	-0.00	-0.01 – 0.00	-0.00	-0.01 – 0.00	-0.08	-0.09 – -0.07	-0.08	-0.09 – -0.07	-0.11	-0.12 – -0.10	-0.11	-0.12 – -0.10
base/non-base form	-0.01	-0.02 – 0.00	-0.01	-0.02 – 0.00	-0.01	-0.02 – 0.00	-0.01	-0.02 – -0.00	-0.01	-0.02 – 0.01	-0.01	-0.03 – 0.00	-0.02	-0.05 – -0.00	-0.03	-0.05 – -0.01
saccade length	0.01	0.01 – 0.01	0.01	0.01 – 0.01	0.01	0.01 – 0.01	0.01	0.01 – 0.01	0.01	0.01 – 0.01	0.01	0.01 – 0.01	0.01	0.01 – 0.01	0.01	0.01 – 0.01
Observations	55717		55717		64428		64428		68633		68633		68633		68633	
Bayes R^2 / Standard Error	0.187 / 0.003		0.186 / 0.003		0.023 / 0.159		0.021 / 0.159		0.168 / 0.002		0.168 / 0.002		0.185 / 0.002		0.185 / 0.002	

	SFD (corpus on cloze)		SFD (cloze on corpus)		FFD (corpus on cloze)		FFD (cloze on corpus)		GD (corpus on cloze)		GD (cloze on corpus)		TT (corpus on cloze)		TT (cloze on corpus)	
Intercept	-0.98	-2.48 – 0.48	0.98	-0.30 – 2.25	-1.00	-2.43 – 0.40	0.78	-0.43 – 1.95	-4.41	-6.47 – -2.07	-0.50	-2.46 – 1.51	-10.05	-13.90– -6.17	-3.21	-6.57 – 0.17
n lexical probability	**-0.40**	**-0.69 – -0.11**	-0.43	-0.97 – 0.11	**-0.39**	**-0.68 – -0.12**	-0.48	-1.03 – 0.08	**-2.33**	**-2.78 – -1.88**	**-3.16**	**-4.02 – -2.28**	**-4.28**	**-5.03 – -3.52**	**-6.12**	**-7.61 – -4.66**
n+1 lexical probability	0.07	-0.20 – 0.34	0.58	0.10 – 1.05	0.07	-0.20 – 0.34	**0.53**	**0.08 – 0.96**	-0.04	-0.48 – 0.40	0.21	-0.50 – 0.96	**-0.95**	**-1.68 – -0.23**	**-1.27**	**-2.52 – -0.08**
n-1 lexical probability	-0.20	-0.47 – 0.08	0.22	-0.30 – 0.77	-0.20	-0.45 – 0.06	0.15	-0.37 – 0.65	0.19	-0.22 – 0.63	**0.98**	**0.12 – 1.86**	-0.07	-0.78 – 0.67	0.73	-0.66 – 2.15
