# Supplementary material for: Morphosyntactic but not lexical corpus-based probabilities can substitute for cloze probabilities in reading experiments
Source: PLoS One. 2021 Jan 28;16(1):e0246133. doi: 10.1371/journal.pone.0246133 (PMC7842903; doi:10.1371/journal.pone.0246133)
Supplement: S3 Table — (PDF) [file pone.0246133.s003.pdf]

**S3 Table. Summaries of model fits with either cloze or corpus-based word class probabilities.**

| | **SFD (cloze probability)** | | **SFD (corpus probability)** | | **FFD (cloze probability)** | | **FFD (corpus probability)** | | **GD (cloze probability)** | | **GD (corpus probability)** | | **TT (cloze probability)** | | **TT (corpus probability)** | |
|---|---|---|---|---|---|---|---|---|---|---|---|---|---|---|---|---|
| *Predictors* | *Estimates* | *HDI (95%)* | *Estimates* | *HDI (95%)* | *Estimates* | *HDI (95%)* | *Estimates* | *HDI (95%)* | *Estimates* | *HDI (95%)* | *Estimates* | *HDI (95%)* | *Estimates* | *HDI (95%)* | *Estimates* | *HDI (95%)* |
| Intercept | 5.43 | 5.39 – 5.46 | 5.40 | 5.36 – 5.44 | 5.42 | 5.39 – 5.45 | 5.39 | 5.35 – 5.42 | 5.54 | 5.50 – 5.58 | 5.49 | 5.45 – 5.54 | 5.70 | 5.64 – 5.76 | 5.59 | 5.52 – 5.65 |
| frequency | -0.02 | -0.03 – -0.02 | -0.02 | -0.03 – -0.02 | -0.02 | -0.03 – -0.02 | -0.02 | -0.02 – -0.01 | -0.03 | -0.04 – -0.02 | -0.03 | -0.03 – -0.02 | -0.05 | -0.06 – -0.04 | -0.03 | -0.04 – -0.02 |
| length | -0.00 | -0.00 – 0.00 | 0.00 | -0.00 – 0.00 | -0.00 | -0.00 – 0.00 | -0.00 | -0.00 – 0.00 | 0.02 | 0.02 – 0.02 | 0.02 | 0.02 – 0.02 | 0.03 | 0.02 – 0.03 | 0.03 | 0.02 – 0.03 |
| n+1 length | -0.03 | -0.03 – -0.02 | -0.03 | -0.03 – -0.02 | -0.02 | -0.03 – -0.02 | -0.02 | -0.03 – -0.02 | -0.04 | -0.04 – -0.03 | -0.03 | -0.04 – -0.03 | -0.04 | -0.05 – -0.03 | -0.04 | -0.05 – -0.03 |
| n+1 frequency | -0.02 | -0.02 – -0.01 | -0.02 | -0.02 – -0.01 | -0.01 | -0.02 – -0.01 | -0.01 | -0.02 – 0.01 | -0.02 | -0.02 – -0.01 | -0.02 | -0.02 – -0.01 | -0.02 | -0.03 – -0.02 | -0.02 | -0.03 – -0.01 |
| n-1 length | 0.00 | -0.00 – 0.01 | 0.00 | -0.00 – 0.01 | 0.00 | -0.00 – 0.01 | 0.00 | -0.00 – 0.01 | -0.00 | -0.01 – 0.01 | -0.00 | -0.01 – 0.01 | -0.01 | -0.02 – 0.00 | -0.01 | -0.02 – 0.00 |
| n-1 frequency | -0.01 | -0.01 – -0.00 | -0.00 | -0.01 – -0.00 | -0.01 | -0.01 – -0.00 | -0.00 | -0.01 – -0.00 | -0.00 | -0.01 – 0.00 | -0.00 | -0.01 – 0.00 | -0.00 | -0.01 – 0.00 | -0.00 | -0.01 – 0.01 |
| landing position | -0.01 | -0.02 – -0.01 | -0.01 | -0.02 – -0.01 | -0.00 | -0.01 – 0.00 | -0.00 | -0.01 – 0.00 | -0.08 | -0.09 – -0.07 | -0.08 | -0.09 – -0.07 | -0.11 | -0.12 – -0.10 | -0.11 | -0.12 – -0.10 |
| saccade length | 0.01 | 0.01 – 0.01 | 0.01 | 0.01 – 0.01 | 0.01 | 0.01 – 0.01 | 0.01 | 0.01 – 0.01 | 0.01 | 0.01 – 0.01 | 0.01 | 0.01 – 0.01 | 0.01 | 0.01 – 0.01 | 0.01 | 0.01 – 0.01 |
| base/non-base form | -0.01 | -0.02 – 0.00 | -0.01 | -0.02 – 0.00 | -0.01 | -0.02 – 0.00 | -0.01 | -0.02 – -0.00 | -0.01 | -0.02 – 0.01 | -0.01 | -0.03 – 0.00 | -0.03 | -0.05 – -0.01 | -0.03 | -0.05 – -0.01 |
| n lexical probability | -0.00 | -0.01 – 0.00 | **-0.00** | **-0.01 – -0.00** | **-0.01** | **-0.01 – -0.00** | **-0.00** | **-0.01 – -0.00** | **-0.01** | **-0.02 – -0.00** | **-0.01** | **-0.02 – -0.01** | **-0.02** | **-0.03 – -0.01** | **-0.02** | **-0.03 – -0.02** |
| n+1 lexical probability | **0.01** | **0.01 – 0.02** | **0.00** | **0.00 – 0.01** | **0.01** | **0.01 – 0.02** | **0.00** | **0.00 – 0.01** | **0.01** | **0.01 – 0.02** | 0.00 | -0.00 – 0.01 | 0.00 | -0.00 – 0.01 | -0.00 | -0.01 – 0.00 |
| n-1 lexical probability | **0.01** | **0.01 – 0.01** | 0.00 | -0.00 – 0.00 | **0.01** | **0.00 – 0.01** | 0.00 | -0.00 – 0.00 | **0.01** | **0.00 – 0.02** | 0.00 | -0.00 – 0.00 | 0.01 | -0.00 – 0.01 | -0.00 | -0.01 – 0.00 |
| n word class probability | **-0.00** | **-0.01 – -0.00** | -0.00 | -0.01 – 0.00 | -0.00 | -0.01 – 0.00 | -0.00 | -0.01 – 0.00 | **-0.01** | **-0.01 – -0.00** | -0.00 | -0.01 – 0.01 | **-0.01** | **-0.02 – -0.00** | -0.00 | -0.01 – 0.01 |
| n+1 word class probability | 0.00 | -0.00 – 0.00 | 0.00 | -0.00 – 0.01 | -0.00 | -0.00 – 0.00 | 0.00 | -0.00 – 0.01 | 0.00 | -0.00 – 0.01 | 0.00 | -0.00 – 0.01 | 0.00 | -0.00 – 0.01 | 0.01 | -0.00 – 0.02 |
| Observations | 55497 | | 55497 | | 64169 | | 64169 | | 68339 | | 68339 | | 68339 | | 68339 | |
| Bayes $R^2$ / Standard Error | 0.187 / 0.003 | | 0.187 / 0.003 | | 0.023 / 0.160 | | 0.021 / 0.159 | | 0.168 / 0.002 | | 0.168 / 0.002 | | 0.185 / 0.002 | | 0.185 / 0.002 | |

| | **SFD (corpus on cloze)** | | **SFD (cloze on corpus)** | | **FFD (corpus on cloze)** | | **FFD (cloze on corpus)** | | **GD (corpus on cloze)** | | **GD (cloze on corpus)** | | **TT (corpus on cloze)** | | **TT (cloze on corpus)** | |
|---|---|---|---|---|---|---|---|---|---|---|---|---|---|---|---|---|
| Intercept | -1.11 | -2.70 – 0.41 | 0.97 | -0.42 – 2.34 | -1.16 | -2.65 – 0.31 | 0.85 | -0.52 – 2.20 | -4.79 | -7.26 – -2.37 | -0.62 | -2.77 – 1.57 | -10.71 | -14.74– -6.61 | -3.84 | -7.48 – -0.03 |
| n lexical probability | **-0.46** | **-0.79 – -0.15** | -0.50 | -1.14 – 0.11 | **-0.46** | **-0.78 – -0.14** | -0.52 | -1.13 – 0.09 | **-2.59** | **-3.10 – -2.07** | **-3.25** | **-4.23 – -2.27** | **-4.66** | **-5.55 – -3.78** | **-6.13** | **-7.86 – -4.45** |
| n+1 lexical probability | 0.06 | -0.26 – 0.40 | **0.62** | **0.06 – 1.18** | 0.04 | -0.28 – 0.36 | **0.58** | **0.07 – 1.12** | -0.01 | -0.54 – 0.52 | 0.21 | -0.62 – 1.05 | **-0.96** | **-1.88 – -0.06** | **-1.60** | **-3.01 – -0.19** |
| n-1 lexical probability | -0.21 | -0.49 – 0.06 | 0.26 | -0.26 – 0.80 | -0.20 | -0.45 – 0.06 | 0.20 | -0.32 – 0.71 | 0.18 | -0.26 – 0.62 | **1.06** | **0.26 – 1.88** | -0.08 | -0.81 – 0.65 | 0.82 | -0.58 – 2.17 |
| n word class probability | 0.38 | -0.41 – 1.19 | 0.17 | -0.36 – 0.67 | 0.39 | -0.32 – 1.13 | 0.12 | -0.37 – 0.60 | **1.24** | **0.07 – 2.40** | 0.30 | -0.51 – 1.09 | 1.86 | -0.21 – 3.79 | 0.27 | -1.08 – 1.59 |
| n+1 word class probability | 0.08 | -0.66 – 0.79 | -0.07 | -0.59 – 0.46 | 0.11 | -0.56 – 0.82 | -0.11 | -0.61 – 0.38 | 0.02 | -1.10 – 1.18 | 0.03 | -0.76 – 0.84 | 0.06 | -1.93 – 2.02 | 0.60 | -0.83 – 1.97 |
