# Supplementary material for: Morphosyntactic but not lexical corpus-based probabilities can substitute for cloze probabilities in reading experiments
Source: PLoS One. 2021 Jan 28;16(1):e0246133. doi: 10.1371/journal.pone.0246133 (PMC7842903; doi:10.1371/journal.pone.0246133)
Supplement: S4 Table — (PDF) [file pone.0246133.s004.pdf]

S4 Table. Summaries of model fits with either cloze or corpus-based morphological probabilities for nouns.

	SFD (cloze probability)		SFD (corpus probability)		FFD (cloze probability)		FFD (corpus probability)		GD (cloze probability)		GD (corpus probability)		TT (cloze probability)		TT (corpus probability)	
Predictors	*Estimates*	*HDI (95%)*	*Estimates*	*HDI (95%)*	*Estimates*	*HDI (95%)*	*Estimates*	*HDI (95%)*	*Estimates*	*HDI (95%)*	*Estimates*	*HDI (95%)*	*Estimates*	*HDI (95%)*	*Estimates*	*HDI (95%)*
Intercept	5.44	5.38 – 5.48	5.42	5.37 – 5.49	5.42	5.37 – 5.47	5.41	5.35 – 5.46	5.54	5.47 – 5.60	5.51	5.43 – 5.58	5.67	5.58 – 5.76	5.58	5.48 – 5.68
frequency	-0.03	-0.04 – -0.02	-0.03	-0.04 – -0.02	-0.03	-0.04 – -0.02	-0.03	-0.04 – -0.02	-0.04	-0.05 – -0.02	-0.04	-0.05 – -0.02	-0.05	-0.07 – -0.03	-0.04	-0.06 – -0.02
length	-0.00	-0.00 – 0.00	-0.00	-0.00 – 0.00	-0.00	-0.00 – 0.00	-0.00	-0.00 – 0.00	0.02	0.01 – 0.02	0.02	0.01 – 0.02	0.02	0.02 – 0.03	0.02	0.02 – 0.03
n+1 length	-0.02	-0.03 – -0.01	-0.02	-0.03 – -0.01	-0.02	-0.03 – -0.01	-0.02	-0.03 – -0.01	-0.02	-0.04 – -0.01	-0.02	-0.04 – -0.00	-0.03	-0.06 – -0.01	-0.03	-0.05 – -0.01
n+1 frequency	-0.01	-0.02 – -0.00	-0.01	-0.02 – 0.00	-0.01	-0.02 – -0.00	-0.01	-0.01 – 0.00	-0.01	-0.02 – 0.00	-0.01	-0.02 – 0.00	-0.02	-0.03 – -0.00	-0.01	-0.03 – 0.00
n-1 length	0.01	-0.00 – 0.02	0.01	-0.01 – 0.02	0.01	-0.01 – 0.02	0.00	-0.01 – 0.02	-0.00	-0.02 – 0.01	-0.00	-0.02 – 0.01	-0.01	-0.03 – 0.01	-0.01	-0.03 – 0.01
n-1 frequency	-0.01	-0.02 – -0.00	-0.01	-0.02 – -0.00	-0.01	-0.02 – -0.00	-0.01	-0.02 – -0.00	-0.01	-0.02 – -0.00	-0.01	-0.02 – 0.00	-0.02	-0.03 – -0.00	-0.01	-0.03 – 0.00
landing position	-0.06	-0.07 – -0.04	-0.06	-0.07 – -0.04	-0.04	-0.06 – 0.03	-0.04	-0.06 – -0.03	-0.14	-0.15 – -0.12	-0.14	-0.15 – -0.12	-0.17	-0.19 – -0.15	-0.17	-0.20 – -0.16
saccade length	0.01	0.01 – 0.01	0.01	0.01 – 0.01	0.01	0.01 – 0.01	-0.01	0.01 – 0.01	0.01	0.01 – 0.01	0.01	0.01 – 0.01	0.01	0.01 – 0.01	0.01	0.01 – 0.01
base/non-base form	-0.01	-0.03 – 0.01	-0.01	-0.03 – 0.01	-0.01	-0.03 – 0.01	-0.01	-0.03 – 0.01	-0.00	-0.03 – 0.02	-0.01	-0.04 – 0.02	-0.01	-0.05 – 0.03	-0.03	-0.07 – 0.00
n lexical probability	**-0.02**	**-0.02 – -0.01**	-0.00	-0.01 – 0.00	**-0.01**	**-0.02 – -0.01**	-0.00	-0.01 – 0.00	**-0.02**	**-0.04 – -0.01**	**-0.01**	**-0.02 – -0.00**	**-0.04**	**-0.06 – -0.02**	**-0.02**	**-0.03 – -0.02**
n+1 lexical probability	**0.01**	**0.00 – 0.02**	-0.00	-0.01 – 0.01	0.01	-0.00 – 0.02	-0.00	-0.01 – 0.00	0.01	-0.01 – 0.02	-0.00	-0.01 – 0.01	0.00	-0.02 – 0.02	-0.01	-0.02 – 0.00
n-1 lexical probability	**0.01**	**0.00 – 0.02**	0.00	-0.00 – 0.01	**0.01**	**0.00 – 0.02**	0.00	-0.00 – 0.01	0.01	-0.00 – 0.02	0.00	-0.00 – 0.01	0.00	-0.01 – 0.02	-0.00	-0.01 – 0.01
n word class probability	0.01	-0.01 – 0.02	0.01	-0.01 – 0.03	0.01	-0.00 – 0.02	0.01	-0.01 – 0.03	0.00	-0.01 – 0.02	0.01	-0.02 – 0.03	0.01	-0.01 – 0.03	0.01	-0.02 – 0.05
n+1 word class probability	0.00	-0.00 – 0.01	0.01	0.00 – 0.02	0.00	-0.00 – 0.01	**0.01**	**0.00 – 0.02**	0.00	-0.01 – 0.01	0.02	0.00 – 0.03	0.01	-0.00 – 0.02	0.03	0.01 – 0.05
n gender probability	-0.01	-0.01 – 0.00	-0.01	-0.03 – 0.00	-0.01	-0.01 – 0.00	-0.01	-0.02 – 0.01	-0.01	-0.02 – 0.00	-0.02	-0.04 – 0.00	**-0.02**	**-0.04 – -0.00**	-0.02	-0.05 – 0.00
n case probability	-0.00	-0.01 – 0.01	0.01	-0.01 – 0.03	0.00	-0.01 – 0.01	0.01	-0.01 – 0.03	-0.00	-0.02 – 0.01	0.01	-0.01 – 0.04	-0.01	-0.03 – 0.01	0.01	-0.02 – 0.05
n number probability	-0.00	-0.01 – 0.01	**-0.02**	**-0.04 – -0.00**	-0.00	-0.02 – 0.01	**-0.02**	**-0.04 – -0.00**	-0.00	-0.02 – 0.01	-0.02	-0.04 – 0.00	0.01	-0.02 – 0.03	-0.02	-0.05 – 0.01
Observations	19652		19652		22510		22510		23628		23628		23628		23628	
Bayes R^2 / Standard Error	0.186 / 0.004		0.186 / 0.004		0.024 / 0.158		0.023 / 0.158		0.167 / 0.004		0.167 / 0.004		0.177 / 0.004		0.177 / 0.004	
	**SFD (corpus on cloze)**		**SFD (cloze on corpus)**		**FFD (corpus on cloze)**		**FFD (cloze on corpus)**		**GD (corpus on cloze)**		**GD (cloze on corpus)**		**TT (corpus on cloze)**		**TT (cloze on corpus)**	
Intercept	-0.18	-3.42 – 3.14	0.64	-2.15 – 3.62	-0.29	-3.34 – 2.74	0.39	-2.44 – 3.22	-1.50	-6.48 – 3.40	0.44	-3.92 – 4.76	-6.44	-14.83 – 1.63	-2.78	-10.50 – 4.62
n lexical probability	-0.47	-1.07 – 0.12	-0.91	-1.92 – 0.13	-0.39	-0.96 – 0.20	-0.80	-1.83 – 0.17	**-1.68**	**-2.55 – -0.76**	**-2.39**	**-3.93 – -0.82**	**-2.72**	**-4.26 – -1.18**	**-4.98**	**-7.62 – -2.33**
n+1 lexical probability	-0.04	-0.56 – 0.50	0.58	-0.45 – 1.60	-0.04	-0.56 – 0.44	0.44	-0.52 – 1.36	-0.31	-1.13 – 0.48	-0.35	-1.90 – 1.19	-1.35	-2.83 – 0.11	-1.61	-4.25 – 0.98
n-1 lexical probability	0.02	-0.50 – 0.53	0.37	-0.63 – 1.38	-0.08	-0.56 – 0.41	0.18	-0.80 – 1.15	0.61	-0.18 – 1.38	1.25	-0.28 – 2.72	-0.17	-1.50 – 1.18	-0.13	-2.60 – 2.40
n word class probability	0.38	-1.99 – 2.71	0.52	-0.75 – 1.78	0.21	-2.02 – 2.50	0.43	-0.79 – 1.68	2.58	-1.00 – 6.01	0.24	-1.70 – 2.24	5.27	-0.94 – 11.50	2.03	-1.34 – 5.35
n+1 word class probability	0.58	-0.65 – 1.79	0.12	-0.84 – 1.03	0.48	-0.68 – 1.64	0.06	-0.82 – 0.92	0.97	-1.00 – 2.93	0.30	-1.10 – 1.73	1.63	-1.64 – 5.04	0.77	-1.61 – 3.18
n gender probability	0.02	-1.80 – 1.84	0.23	-0.77 – 1.26	0.08	-1.68 – 1.87	0.11	-0.88 – 1.09	-1.85	-4.66 – 0.98	-0.44	-2.02 – 1.10	-4.18	-8.89 – 0.64	-2.35	-5.05 – 0.44
n case probability	0.74	-1.54 – 3.09	-0.38	-1.75 – 1.01	0.68	-1.51 – 2.95	-0.26	-1.58 – 1.01	0.31	-3.15 – 3.72	-0.38	-2.47 – 1.65	-1.33	-7.48 – 5.06	-1.48	-4.95 – 1.96
n number probability	-1.08	-3.17 – 0.93	0.06	-1.37 – 1.47	-1.07	-3.05 – 0.89	-0.05	-1.36 – 1.34	-0.72	-3.85 – 2.44	0.35	-1.82 – 2.48	-0.55	-5.85 – 4.68	0.42	-3.09 – 3.98
