# Supplementary material for: Morphosyntactic but not lexical corpus-based probabilities can substitute for cloze probabilities in reading experiments
Source: PLoS One. 2021 Jan 28;16(1):e0246133. doi: 10.1371/journal.pone.0246133 (PMC7842903; doi:10.1371/journal.pone.0246133)
Supplement: S5 Table — (PDF) [file pone.0246133.s005.pdf]

S5 Table. Summaries of model fits with either cloze or corpus-based morphological probabilities for verbs in present and future tenses.

	**SFD (cloze probability)**		**SFD (corpus probability)**		**FFD (cloze probability)**		**FFD (corpus probability)**		**GD (cloze probability)**		**GD (corpus probability)**		**TT (cloze probability)**		**TT (corpus probability)**	
Predictors	*Estimates*	*HDI (95%)*	*Estimates*	*HDI (95%)*	*Estimates*	*HDI (95%)*	*Estimates*	*HDI (95%)*	*Estimates*	*HDI (95%)*	*Estimates*	*HDI (95%)*	*Estimates*	*HDI (95%)*	*Estimates*	*HDI (95%)*
Intercept	5.46	5.36 – 5.57	5.39	5.27 – 5.52	5.44	5.34 – 5.54	5.37	5.25 – 5.49	5.57	5.44 – 5.70	5.50	5.33 – 5.67	5.71	5.48 – 5.93	5.59	5.31 – 5.86
frequency	-0.05	-0.08 – -0.02	-0.04	-0.07 – -0.00	-0.05	-0.08 – -0.02	-0.04	-0.07 – -0.00	-0.08	-0.12 – -0.04	-0.06	-0.10 – -0.01	-0.12	-0.19 – -0.05	-0.08	-0.17 – -0.01
length	-0.01	-0.02 – 0.00	-0.01	-0.02 – 0.00	-0.01	-0.02 – 0.00	-0.01	-0.02 – 0.00	0.02	0.00 – 0.03	0.02	0.00 – 0.03	0.02	-0.00 – 0.04	0.02	-0.01 – 0.04
n+1 length	0.01	-0.01 – 0.04	0.01	-0.01 – 0.04	0.02	-0.01 – 0.04	0.02	-0.01 – 0.04	0.02	-0.01 – 0.05	0.02	-0.02 – 0.05	0.04	-0.01 – 0.10	0.03	-0.03 – 0.09
n+1 frequency	0.00	-0.02 – 0.03	0.01	-0.02 – 0.03	0.01	-0.01 – 0.03	0.01	-0.01 – 0.03	0.02	-0.01 – 0.04	0.02	-0.02 – 0.05	0.01	-0.03 – 0.06	0.02	-0.04 – 0.07
n-1 length	-0.01	-0.04 – 0.02	-0.00	-0.03 – 0.03	-0.01	-0.03 – 0.02	0.00	-0.02 – 0.03	0.00	-0.03 – 0.04	0.01	-0.03 – 0.05	0.03	-0.04 – 0.09	0.05	-0.02 – 0.11
n-1 frequency	-0.04	-0.06 – -0.01	-0.03	-0.06 – -0.01	-0.03	-0.05 – -0.00	-0.03	-0.05 – -0.00	-0.03	-0.06 – 0.00	-0.03	-0.06 – 0.01	-0.02	-0.07 – 0.04	-0.01	-0.07 – 0.05
landing position	-0.03	-0.06 – 0.00	-0.03	-0.06 – 0.00	0.01	-0.02 – 0.04	0.01	-0.02 – 0.04	-0.19	-0.24 – -0.15	-0.19	-0.24 – -0.15	-0.24	-0.30 – -0.18	-0.24	-0.30 – -0.18
saccade length	0.01	0.01 – 0.01	0.01	0.01 – 0.01	0.01	0.00 – 0.01	0.01	0.00 – 0.01	0.00	0.00 – 0.01	0.00	0.00 – 0.01	0.00	-0.00 – 0.00	0.00	-0.00 – 0.00
base/non-base form	-0.00	-0.20 – 0.19	0.00	-0.20 – 0.20	0.00	-0.19 – 0.19	-0.00	-0.20 – 0.19	-0.00	-1.01 – 0.96	0.00	-1.02 – 0.93	-0.00	-0.95 – 1.00	-0.00	-1.01 – 0.96
n lexical probability	-0.02	-0.05 – 0.01	-0.02	-0.03 – -0.01	-0.02	-0.04 – 0.01	**-0.02**	**-0.03 – -0.00**	-0.02	-0.05 – 0.02	-0.02	-0.04 – -0.00	-0.05	-0.11 – 0.01	-0.05	-0.08 – -0.02
n+1 lexical probability	0.00	-0.03 – 0.03	-0.00	-0.02 – 0.01	-0.00	-0.03 – 0.02	-0.01	-0.02 – 0.01	-0.02	-0.05 – 0.02	-0.01	-0.02 – 0.01	-0.04	-0.10 – 0.03	-0.02	-0.05 – 0.01
n-1 lexical probability	0.02	-0.00 – 0.04	0.01	-0.01 – 0.02	0.02	-0.00 – 0.04	0.01	-0.00 – 0.02	0.02	-0.01 – 0.05	0.01	-0.01 – 0.02	0.02	-0.03 – 0.07	0.01	-0.01 – 0.04
n word class probability	0.01	-0.02 – 0.04	0.03	-0.02 – 0.08	0.01	-0.02 – 0.04	0.03	-0.02 – 0.07	-0.00	-0.04 – 0.04	0.02	-0.04 – 0.09	-0.01	-0.07 – 0.06	0.02	-0.10 – 0.14
n+1 word class probability	0.01	-0.01 – 0.04	0.01	-0.02 – 0.05	0.01	-0.01 – 0.04	0.01	-0.02 – 0.04	0.01	-0.02 – 0.04	-0.00	-0.05 – 0.04	0.02	-0.04 – 0.07	0.01	-0.07 – 0.09
n tense probability	-0.00	-0.03 – 0.02	0.01	-0.03 – 0.04	-0.01	-0.03 – 0.02	0.00	-0.03 – 0.04	**-0.03**	**-0.07 – -0.00**	-0.03	-0.08 – 0.02	**-0.09**	**-0.15 – -0.03**	-0.08	-0.17 – 0.01
n number probability	-0.01	-0.03 – 0.02	0.03	-0.02 – 0.08	-0.01	-0.04 – 0.02	0.02	-0.03 – 0.07	0.01	-0.02 – 0.04	0.04	-0.03 – 0.12	0.00	-0.05 – 0.07	0.01	-0.11 – 0.14
n person probability	-0.01	-0.04 – 0.02	-0.02	-0.06 – 0.02	-0.01	-0.03 – 0.02	-0.02	-0.05 – 0.02	0.00	-0.03 – 0.04	-0.01	-0.06 – 0.05	0.06	-0.01 – 0.12	0.06	-0.03 – 0.16
Observations	3207		3207		3979		3979		4161		4161		4161		4161	
Bayes R^2 / Standard Error	0.230 / 0.012		0.230 / 0.011		0.038 / 0.195		0.040 / 0.195		0.210 / 0.009		0.210 / 0.009		0.276 / 0.012		0.277 / 0.012	

	**SFD (corpus on cloze)**		**SFD (cloze on corpus)**		**FFD (corpus on cloze)**		**FFD (cloze on corpus)**		**GD (corpus on cloze)**		**GD (cloze on corpus)**		**TT (corpus on cloze)**		**TT (cloze on corpus)**	
Intercept	-4.26	-11.04 – 2.28	-0.80	-8.21 – 6.77	-3.55	-9.89 – 2.98	-1.15	-8.16 – 5.67	-12.57	-26.20 – 0.87	-8.57	-22.63 – 5.45	-20.89	-41.87 – 0.55	-13.92	-36.76 – 8.37
n lexical probability	-1.16	-2.67 – 0.36	-1.17	-4.70 – 2.36	-1.04	-2.42 – 0.32	-0.95	-4.26 – 2.32	**-4.67**	**-7.61 – -1.79**	-2.63	-9.53 – 4.03	**-7.20**	**-11.85– -2.57**	-7.42	-17.96 – 3.50
n+1 lexical probability	-0.17	-1.30 – 1.01	0.04	-3.29 – 3.41	-0.11	-1.17 – 0.95	-0.28	-3.28 – 2.78	0.03	-2.21 – 2.29	-1.96	-8.35 – 4.31	-1.79	-5.42 – 1.87	-3.28	-13.44 – 6.56
n-1 lexical probability	-0.05	-1.13 – 1.00	0.22	-2.27 – 2.63	-0.00	-1.00 – 0.98	0.08	-2.28 – 2.40	0.48	-1.50 – 2.42	-1.24	-5.85 – 3.45	1.95	-1.48 – 5.29	0.03	-8.17 – 7.87
n word class probability	0.37	-5.35 – 6.18	0.56	-3.10 – 4.19	0.22	-5.29 – 5.56	0.55	-2.69 – 3.85	6.42	-5.23 – 17.76	-0.81	-7.53 – 5.80	2.59	-15.14–20.70	-3.42	-14.60 – 7.62
n+1 word class probability	-0.24	-3.86 – 3.12	0.12	-2.40 – 2.70	-0.59	-3.77 – 2.61	0.08	-2.28 – 2.43	-0.97	-7.83 – 5.90	-0.23	-5.01 – 4.70	5.51	-5.13 – 15.94	1.05	-6.51 – 8.57
n tense probability	0.61	-4.17 – 5.38	0.42	-2.67 – 3.36	0.83	-3.57 – 5.07	0.01	-2.80 – 2.88	-4.44	-13.58 – 4.81	**-6.56**	**-12.24– -0.75**	-12.82	-27.61 – 1.65	**-10.01**	**-19.32– -0.96**
n number probability	2.44	-4.43 – 9.15	-0.63	-3.78 – 2.58	1.51	-4.57 – 7.69	-0.65	-3.60 – 2.29	-1.97	-15.21– 10.99	0.06	-5.74 – 6.05	-7.55	-28.21–13.87	-1.19	-11.04 – 8.18
n person probability	-0.94	-5.87 – 3.95	-0.68	-3.83 – 2.45	-0.76	-5.14 – 3.64	-0.33	-3.20 – 2.63	1.83	-7.33 – 10.97	4.19	-1.47 – 9.87	12.71	-1.69 – 27.20	8.53	-0.48 – 18.10
