# Supplementary material for: Morphosyntactic but not lexical corpus-based probabilities can substitute for cloze probabilities in reading experiments
Source: PLoS One. 2021 Jan 28;16(1):e0246133. doi: 10.1371/journal.pone.0246133 (PMC7842903; doi:10.1371/journal.pone.0246133)
Supplement: S6 Table — (PDF) [file pone.0246133.s006.pdf]

S6 Table. Summaries of model fits with either cloze or corpus-based morphological probabilities for verbs in past tense.

	**SFD (cloze probability)**		**SFD (corpus probability)**		**FFD (cloze probability)**		**FFD (corpus probability)**		**GD (cloze probability)**		**GD (corpus probability)**		**TT (cloze probability)**		**TT (corpus probability)**	
Predictors	*Estimates*	*HDI (95%)*	*Estimates*	*HDI (95%)*	*Estimates*	*HDI (95%)*	*Estimates*	*HDI (95%)*	*Estimates*	*HDI (95%)*	*Estimates*	*HDI (95%)*	*Estimates*	*HDI (95%)*	*Estimates*	*HDI (95%)*
Intercept	5.47	5.38 – 5.56	5.39	5.26 – 5.51	5.46	5.37 – 5.54	5.37	5.26 – 5.49	5.55	5.44 – 5.65	5.50	5.36 – 5.65	5.75	5.60 – 5.89	5.59	5.40 – 5.80
frequency	-0.02	-0.04 – -0.00	-0.03	-0.06 – -0.01	-0.02	-0.04 – -0.00	-0.03	-0.05 – -0.01	-0.03	-0.06 – -0.01	-0.04	-0.07 – -0.01	-0.04	-0.08 – -0.01	-0.04	-0.08 – -0.00
length	0.00	-0.01 – 0.01	-0.00	-0.01 – 0.01	-0.00	-0.01 – 0.01	-0.00	-0.01 – 0.01	0.02	0.01 – 0.04	0.02	0.01 – 0.04	0.02	0.01 – 0.04	0.03	0.01 – 0.04
n+1 length	-0.04	-0.07 – -0.02	-0.04	-0.06 – -0.01	-0.04	-0.06 – -0.01	-0.03	-0.05 – -0.01	-0.06	-0.09 – -0.03	-0.06	-0.09 – -0.02	-0.07	-0.11 – -0.02	-0.06	-0.10 – -0.01
n+1 frequency	-0.01	-0.03 – 0.00	0.00	-0.02 – 0.02	-0.01	-0.03 – 0.00	0.00	-0.02 – 0.02	-0.02	-0.04 – 0.01	-0.01	-0.04 – 0.02	-0.03	-0.06 – 0.00	-0.01	-0.04 – 0.03
n-1 length	0.01	-0.02 – 0.03	0.00	-0.03 – 0.03	0.01	-0.02 – 0.03	0.00	-0.02 – 0.03	0.01	-0.03 – 0.04	-0.00	-0.03 – 0.04	-0.00	-0.04 – 0.04	-0.00	-0.05 – 0.05
n-1 frequency	-0.02	-0.03 – 0.00	-0.01	-0.03 – 0.01	-0.01	-0.03 – 0.01	-0.00	-0.03 – 0.02	-0.00	-0.03 – 0.02	-0.00	-0.03 – 0.03	-0.01	-0.04 – 0.02	-0.00	-0.04 – 0.03
landing position	-0.01	-0.04 – 0.01	-0.01	-0.04 – 0.01	-0.01	-0.03 – 0.01	-0.01	-0.03 – 0.02	-0.10	-0.13 – -0.08	-0.10	-0.13 – -0.08	-0.16	-0.19 – -0.12	-0.16	-0.19 – -0.12
saccade length	0.01	0.01 – 0.01	0.01	0.01 – 0.01	0.01	0.00 – 0.01	0.01	0.01 – 0.01	0.01	0.01 – 0.01	0.01	0.01 – 0.01	0.01	0.00 – 0.01	0.01	0.00 – 0.01
base/non-base form	0.06	-0.07 – 0.18	0.06	-0.06 – 0.18	0.03	-0.09 – 0.15	0.04	-0.08 – 0.16	0.06	-0.14 – 0.24	0.13	-0.05 – 0.31	-0.02	-0.28 – 0.23	0.10	-0.15 – 0.32
n lexical probability	0.00	-0.03 – 0.03	0.00	-0.01 – 0.02	0.01	-0.02 – 0.03	0.00	-0.01 – 0.02	-0.01	-0.04 – 0.02	0.00	-0.02 – 0.02	-0.01	-0.06 – 0.04	-0.01	-0.04 – 0.01
n+1 lexical probability	-0.00	-0.02 – 0.02	-0.01	-0.02 – 0.01	-0.00	-0.02 – 0.02	-0.01	-0.02 – 0.01	-0.01	-0.03 – 0.01	-0.00	-0.02 – 0.01	-0.02	-0.05 – 0.01	-0.01	-0.04 – 0.01
n-1 lexical probability	0.02	0.00 – 0.03	0.00	-0.01 – 0.01	**0.02**	**0.00 – 0.03**	0.00	-0.01 – 0.01	0.02	0.01 – 0.04	0.00	-0.01 – 0.02	0.01	-0.01 – 0.04	-0.00	-0.02 – 0.02
n word class probability	-0.02	-0.05 – 0.01	-0.02	-0.06 – 0.03	-0.02	-0.05 – 0.00	-0.01	-0.06 – 0.03	-0.03	-0.07 – 0.00	-0.01	-0.08 – 0.04	**-0.06**	**-0.11 – -0.01**	-0.04	-0.12 – 0.04
n+1 word class probability	0.01	-0.01 – 0.03	0.02	-0.01 – 0.05	0.01	-0.01 – 0.03	0.02	-0.01 – 0.05	0.00	-0.02 – 0.03	-0.00	-0.04 – 0.03	0.02	-0.02 – 0.05	0.01	-0.04 – 0.06
n tense probability	0.01	-0.02 – 0.04	0.01	-0.02 – 0.04	0.01	-0.01 – 0.04	0.01	-0.02 – 0.04	0.01	-0.03 – 0.04	-0.01	-0.05 – 0.03	0.02	-0.03 – 0.06	0.00	-0.05 – 0.06
n number probability	-0.01	-0.04 – 0.02	-0.01	-0.09 – 0.05	-0.01	-0.04 – 0.02	-0.00	-0.07 – 0.06	0.00	-0.04 – 0.04	0.03	-0.06 – 0.12	-0.00	-0.06 – 0.05	0.06	-0.06 – 0.18
n gender probability	0.02	-0.00 – 0.04	0.02	-0.03 – 0.06	0.01	-0.01 – 0.03	0.01	-0.04 – 0.05	0.01	-0.01 – 0.04	0.01	-0.05 – 0.07	0.01	-0.03 – 0.04	0.00	-0.08 – 0.08
Observations	5567		5567		6581		6581		7007		7007		7007		7007	
Bayes R^2 / Standard Error	0.222 / 0.009		0.222 / 0.009		0.036 / 0.177		0.033 / 0.176		NA		0.192 / 0.007		NA		NA	
	**SFD (corpus on cloze)**		**SFD (cloze on corpus)**		**FFD (corpus on cloze)**		**FFD (cloze on corpus)**		**GD (corpus on cloze)**		**GD (cloze on corpus)**		**TT (corpus on cloze)**		**TT (cloze on corpus)**	
Intercept	-2.74	-9.50 – 4.19	0.70	-4.75 – 6.10	-3.04	-9.74 – 3.29	1.03	-4.53 – 6.42	-8.87	-19.91 – 1.76	-1.60	-10.39 – 7.04	-15.07	-35.04 – 5.02	1.92	-14.41 – 18.13
n lexical probability	-0.23	-1.57 – 1.09	-0.13	-2.56 – 2.35	-0.32	-1.57 – 0.99	0.00	-2.36 – 2.42	**-2.13**	**-4.16 – -0.11**	**-3.81**	**-7.66 – -0.01**	**-4.56**	**-8.30 – -0.83**	-5.40	-12.67 – 1.56
n+1 lexical probability	-0.46	-1.49 – 0.60	-0.06	-2.07 – 1.91	-0.54	-1.52 – 0.43	-0.18	-2.10 – 1.68	-0.44	-2.16 – 1.22	-0.77	-3.66 – 2.12	-2.51	-5.62 – 0.59	-3.42	-8.95 – 2.25
n-1 lexical probability	-0.56	-1.46 – 0.32	0.12	-1.56 – 1.81	-0.50	-1.34 – 0.33	0.14	-1.35 – 1.73	-0.16	-1.48 – 1.19	2.21	-0.25 – 4.72	-0.02	-2.55 – 2.53	2.68	-1.94 – 7.17
n word class probability	1.06	-3.01 – 4.93	-0.69	-3.32 – 1.92	0.92	-2.80 – 4.72	-0.81	-3.50 – 1.78	1.96	-4.20 – 7.99	-0.57	-4.57 – 3.36	4.07	-7.10 – 15.44	-0.85	-8.36 – 6.88
n+1 word class probability	0.39	-2.46 – 3.41	0.35	-1.96 – 2.59	0.71	-2.17 – 3.60	0.46	-1.66 – 2.62	-0.65	-5.38 – 4.26	0.30	-3.08 – 3.57	1.05	-7.55 – 9.87	3.13	-3.40 – 9.47
n tense probability	0.53	-2.13 – 3.24	0.45	-1.95 – 2.92	0.18	-2.60 – 2.81	0.30	-2.10 – 2.74	-3.91	-8.01 – 0.39	-2.47	-6.26 – 1.14	-5.44	-13.25 – 2.56	-4.38	-11.23 – 2.81
n number probability	0.30	-6.84 – 7.62	-0.24	-3.54 – 3.08	0.64	-6.31 – 7.36	-0.34	-3.52 – 2.81	8.41	-2.91 – 19.83	0.55	-4.52 – 5.77	4.46	-17.34–26.72	-3.16	-13.17 – 6.40
n gender probability	-1.09	-5.56 – 3.28	0.12	-1.87 – 2.12	-0.83	-5.15 – 3.44	0.22	-1.73 – 2.13	-2.00	-9.21 – 4.92	0.57	-2.58 – 3.77	-0.19	-13.38–13.13	0.73	-5.18 – 6.38
